# Inhibition of Tyrosinase and Melanogenesis by Carboxylic Acids: Mechanistic Insights and Safety Evaluation

**DOI:** 10.3390/molecules30071642

**Published:** 2025-04-07

**Authors:** Yu-Pei Chen, Mingyu Li, Zirong Liu, Jinxiong Wu, Fangfang Chen, Shudi Zhang

**Affiliations:** 1 The School of Public Health, Fujian Medical University, Fuzhou 350122, China; 2230220214@fjmu.edu.cn; 2 The School of Public Health and Medical Technology, Xiamen Medical College, Xiamen 361023, China; 18030137072@163.com (Z.L.); 18559120270@163.com (J.W.); 200700010183@xmmc.edu.cn (F.C.); zsd@xmmc.edu.cn (S.Z.); 3 Engineering Research Center of Natural Cosmeceuticals College of Fujian Province, Xiamen Medical College, Xiamen 361023, China

**Keywords:** carboxylic acid, 3-phenyllactic acid, L-pyroglutamic acid, tyrosinase, DOPA auto-oxidation, melanin

## Abstract

It is well established that certain carboxylic acid compounds can effectively inhibit tyrosinase activity. This study investigated the mechanisms by which four carboxylic acid compounds—3-phenyllactic acid, lactic acid, L-pyroglutamic acid, and malic acid—inhibit tyrosinase and melanogenesis. IC_50_ values for mushroom tyrosinase inhibition ranged from 3.38 to 5.42 mM, with 3-phenyllactic acid (3.50 mM), lactic acid (5.42 mM), and malic acid (3.91 mM) exhibiting mixed-type inhibition, while L-pyroglutamic acid (3.38 mM) showed competitive inhibition, as determined by enzymatic kinetic analysis. Additionally, the acidification effects of lactic acid, L-pyroglutamic acid, and malic acid contributed to the reduction in tyrosinase activity. Furthermore, all four carboxylic acid compounds effectively inhibited DOPA auto-oxidation (IC_50_ = 0.38–0.66 mM), ranking in potency as follows: malic acid (0.38 mM) > lactic acid (0.57 mM) > 3-phenyllactic acid (0.63 mM) > L-pyroglutamic acid (0.66 mM). These compounds also demonstrated a dose-dependent reduction in melanin production in B16-F10 cells. Proteomic analysis further revealed that these compounds not only inhibit key proteins involved in melanin synthesis, such as tyrosinase, tyrosinase-related protein 1, and tyrosinase-related protein 2, but also potentially modulate other genes associated with melanogenesis and metabolism, including Pmel, Slc45a2, Ctns, Oca2, and Bace2. Network toxicology analysis indicated that these four compounds exhibit a low risk of inducing dermatitis. These findings suggest that these compounds may indirectly regulate melanin-related pathways through multiple mechanisms, highlighting their potential for further applications in cosmetics and pharmaceuticals.

## 1. Introduction

The formation of hyperpigmentation is influenced by a variety of factors, including postinflammatory hyperpigmentation (PIH) [1], ultraviolet (UV) exposure [2], drug-induced pigmentation [3], and hormonal fluctuations (catamenial hyperpigmentation) [4]. Postinflammatory hyperpigmentation primarily occurs due to melanin deposition in response to acute or chronic inflammatory stimuli in the skin [1]. Ultraviolet radiation directly activates melanocytes, promoting melanin synthesis and causing skin darkening or the development of pigmented spots [2]. Additionally, certain medications, such as antiretrovirals, antipsychotics, antimalarials, antibiotics, chemotherapeutic agents, prostaglandin analogs, and heavy metals, may induce generalized or localized hyperpigmentation [3,5,6]. In women, progesterone receptor activity during pregnancy and estrogen level fluctuations throughout the menstrual cycle can disrupt hormonal balance, stimulating melanin production and exacerbating hyperpigmentation [4]. These pigmentation disorders often significantly impair patients’ quality of life. A study assessing 1398 melasma patients using the Melasma Area and Severity Index (MASI) and Melasma Quality of Life (MELASQoL) scales demonstrated that melasma not only causes emotional distress but also negatively impacts social functioning [7].

Melanin is primarily composed of two distinct pigments: pheomelanin (containing sulfur) and eumelanin (brown/black pigment) [8]. Melanogenesis, the process of melanin synthesis, begins with the enzymatic oxidation of tyrosine to L-dopaquinone by tyrosinase. In the presence of cysteine or glutathione, L-dopaquinone undergoes reduction to form pheomelanin. Conversely, in the absence of these antioxidants, it is sequentially catalyzed by tyrosinase, tyrosinase-related protein-1, and tyrosinase-related protein-2 to produce eumelanin. Moreover, Microphthalmia-associated transcription factor (MITF) serves as a central regulator of melanogenesis [9]. As a transcription factor, MITF binds to the promoter regions of genes encoding tyrosinase, TRP-1, and TRP-2, thereby activating their transcriptional expression. The activity of MITF is dynamically modulated by multiple signaling pathways, including the cAMP/PKA, MAPK/ERK, and Wnt/β-catenin pathways [9,10]. Extrinsic stimuli such as UV radiation can activate these pathways, leading to MITF upregulation [10]. This activation subsequently enhances the expression of tyrosinase, tyrosinase-related protein-1, and tyrosinase-related protein-2, ultimately promoting melanin synthesis.

Melanogenesis inhibitors can be classified into multiple categories based on their pharmacological mechanisms and molecular targets [11]. Chemical reducing agents, such as baicalein, rifampicin, and ascorbic acid, inhibit melanin synthesis by reducing dopaquinone [12,13,14], thereby preventing the formation of dopachrome and the subsequent melanin biosynthesis. Another important class of inhibitors comprises thiol compounds, which react with dopaquinone to form colorless products, effectively suppressing melanin synthesis [15]. Additionally, compounds such as hematoxylin and oxyresveratrol exhibit a strong affinity for tyrosinase and inhibit the formation of dopachrome [16,17]. Some compounds, including caffeic acid and its n-nonyl ester, function as suicide substrates for tyrosinase [18]. These compounds inactivate tyrosinase through catalytic reactions, thereby blocking melanin synthesis. Furthermore, certain acidic or alkaline compounds can nonspecifically inhibit the activity of enzymes involved in the melanogenesis pathway, indirectly reducing melanin production. Finally, specific tyrosinase inhibitors are widely used in whitening products due to their ability to directly and selectively inhibit tyrosinase activity. Currently, research on tyrosinase inhibitors is extensive, and these inhibitors can be broadly classified into several classes [19]. These include polyphenols, flavonoids (such as flavones, dihydroflavones, flavonols, isoflavones, flavanones, flavanols, and flavan-3,4-diols), anthocyanidins, curcuminoids, coumarins, chalcones and dihydrochalcones, aurones, phenolic acids, stilbenes, lignans, terpenoid derivatives, quinone derivatives, phenyl derivatives, pyridine, piperidine, pyridinones, and hydroxypyridinone derivatives. Additionally, thiosemicarbazones, thiosemicarbazides, and other thioderivatives, as well as azole and thiazolidine derivatives, are also notable. Other classes include kojic acid analogs, benzaldehyde derivatives, xanthate derivatives, and carboxylic acids.

In our previous studies, several carboxylic acids derived from kefir grains were identified as exhibiting tyrosinase inhibitory activity [20]. These include 3-phenyllactic acid, lactic acid, L-pyroglutamic acid, malic acid, and succinic acid. These carboxylic acids are produced during the fermentation process of kefir grains and are believed to contribute to the melanin-inhibiting properties of kefir fermentation products. The role of succinic acid, a fermentation product of acetic acid bacteria in kefir grains, in melanin inhibition has been previously validated [21]. Notably, phenyllactic acid derived from probiotic metabolism has also demonstrated tyrosinase-inhibitory effects [22]. Therefore, this study aims to further investigate the kinetic analysis of tyrosinase inhibition by the other four carboxylic acids. Additionally, the potential of these acids to inhibit melanogenesis will be evaluated using B16-F10 cells. Their mechanisms of action in melanin inhibition will be elucidated through proteomic analysis. Finally, the potential of these four compounds to induce dermatitis when applied to the skin will be assessed using network toxicology analysis.

## 2. Results

### 2.1. Effect of 3-Phenyllactic Acid, Lactic Acid, L-pyroglutamic Acid, and Malic Acid on Tyrosinase

**Figure 1:**
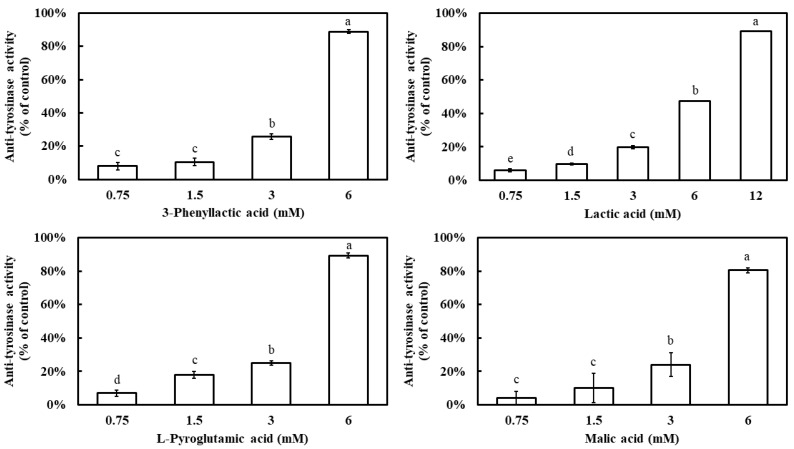
Anti-tyrosinase activity of 3-phenyllactic acid, lactic acid, L-pyroglutamic acid, and malic acid. The columns marked with different letters indicate statistically significant differences (*p* < 0.05). Data are expressed as mean ± standard deviation (S.D.) (*n* = 3).

The inhibitory effects of four carboxylic acids—3-phenyllactic acid, lactic acid, L-pyroglutamic acid, and malic acid—on mushroom tyrosinase activity were evaluated. The results demonstrated that all four compounds exhibited a dose-dependent inhibition pattern, with tyrosinase inhibition rates significantly increasing as their concentrations increased (Figure 1). At a concentration of 6 mM, 3-phenyllactic acid, L-pyroglutamic acid, and malic acid achieved inhibition rates exceeding 80%, while lactic acid showed a lower inhibition rate of 47%. However, when the concentration of lactic acid was increased to 12 mM, its inhibition rate reached 89%. The IC_50_ values for tyrosinase inhibition, calculated using SPSS 27, were determined to be 3.50 mM for 3-phenyllactic acid, 5.42 mM for lactic acid, 3.38 mM for L-pyroglutamic acid, and 3.91 mM for malic acid.

**Figure 2:**
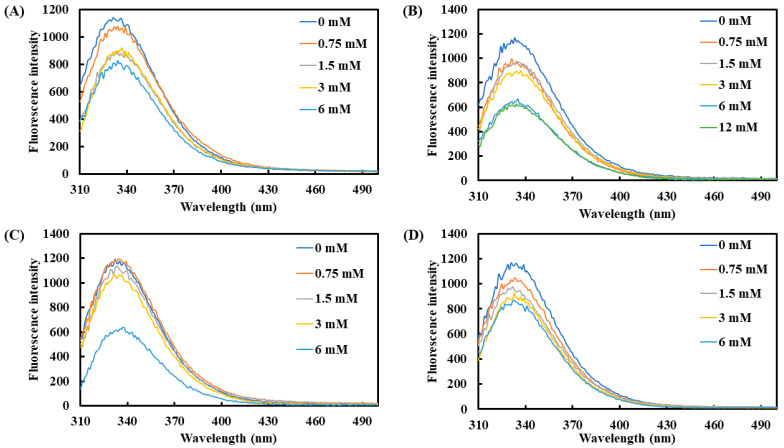
Fluorescence spectra of tyrosinase treated with varying concentrations of (**A**) 3-phenyllactic acid, (**B**) lactic acid, (**C**) L-pyroglutamic acid, and (**D**) malic acid were measured using a microplate reader.

Additionally, tryptophan, tyrosine, and phenylalanine were used as effective fluorescent indicators to monitor changes in protein structure. The average maximum emission wavelength of tyrosinase was observed between 331 and 335 nm (Figure 2). Upon the addition of the four carboxylic acids, the fluorescence intensity of tyrosinase showed a declining trend with increasing concentrations. At 6 mM, lactic acid and L-pyroglutamic acid induced the most pronounced reduction in fluorescence intensity, with decreases of 45.0% and 48.6%, respectively. On the other hand, 3-phenyllactic acid and malic acid also significantly reduced fluorescence intensity by 29.3% and 29.0%, respectively, at 6 mM.

### 2.2. Kinetic Analysis of Tyrosinase by 3-Phenyllactic Acid, Lactic Acid, L-pyroglutamic Acid, and Malic Acid

**Figure 3:**
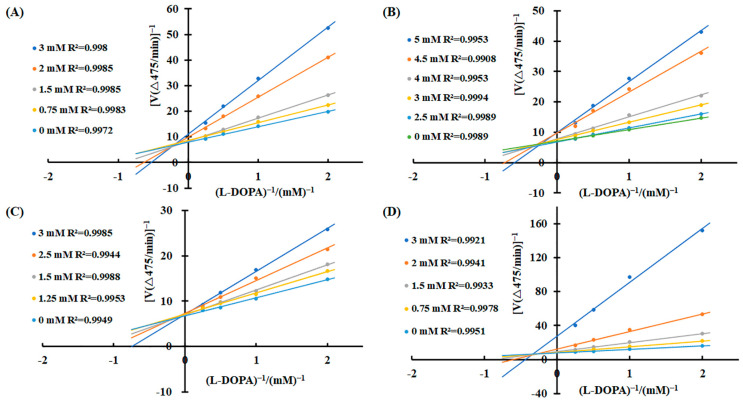
Lineweaver–Burk plots were constructed to analyze the inhibitory effects of 3-phenyllactic acid, lactic acid, L-pyroglutamic acid, and malic acid on tyrosinase activity. Enzymatic kinetic analysis was performed using varying concentrations of (**A**) 3-phenyllactic acid, (**B**) lactic acid, (**C**) L-pyroglutamic acid, and (**D**) malic acid. Kinetic parameters, including the V_max_, K_m_, and K_i_, were determined using GraphPad Prism.

To elucidate the inhibitory mechanisms of the four carboxylic acids—3-phenyllactic acid, lactic acid, L-pyroglutamic acid, and malic acid—against tyrosinase activity, kinetic analyses were performed using the Lineweaver–Burk equation. Double-reciprocal plots were constructed with 1/v as the *Y*-axis and 1/[S] as the *X*-axis. The results revealed distinct inhibition patterns for each compound (Figure 3). For 3-phenyllactic acid, lactic acid, and malic acid, increasing concentrations led to an increase in the Michaelis constant (K_m_) and a concomitant decrease in the maximum reaction rate (V_max_). Specifically, the K_m_ value for 3-phenyllactic acid increased from 0.831 to 2.333, while its V_max_ decreased from 0.526 to 0.409. Similarly, lactic acid exhibited an increase in K_m_ from 0.536 to 2.260 and a reduction in V_max_ from 0.575 to 0.479. Malic acid showed the most pronounced changes, with the K_m_ rising from 0.524 to 4.191 and V_max_ declining from 0.520 to 0.199. The double-reciprocal plots for these three compounds displayed intersecting lines at points neither on the *X*-axis nor the *Y*-axis, indicating a mixed-type inhibition mechanism. In contrast, L-pyroglutamic acid exhibited a different inhibition pattern. While its K_m_ value increased from 0.544 to 1.51, the V_max_ remained relatively stable, fluctuating between 0.554 and 0.601. The double-reciprocal plots for L-pyroglutamic acid showed intersecting lines at the same point on the *Y*-axis (1/V_max_), suggesting a competitive inhibition mechanism. Additionally, the inhibition constants (K_i_) for 3-phenyllactic acid, lactic acid, L-pyroglutamic acid, and malic acid were determined to be 1.569 mM, 2.710 mM, 1.949 mM, and 0.606 mM, respectively. For the mixed-type inhibitors, the inhibition constants (K_IS_) for enzyme–substrate complexes were calculated to be as follows: 8.526 mM for 3-phenyllactic acid, 17.032 mM for lactic acid, and 3.066 mM for malic acid. These findings provide valuable insights into the kinetic behavior and inhibitory mechanisms of these carboxylic acids against tyrosinase activity.

### 2.3. Effect of 3-Phenyllactic Acid, Lactic Acid, L-pyroglutamic Acid, and Malic Acid on pH and L-DOPA Auto-Oxidation

**Figure 4:**
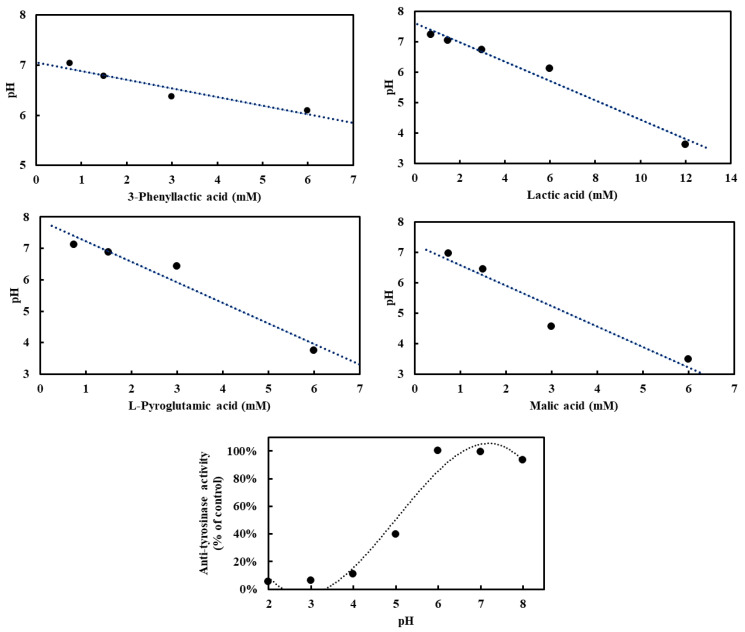
Various concentrations of 3-phenyllactic acid, lactic acid, L-pyroglutamic acid, and malic acid, dissolved in H₂O, were mixed with PBS buffer in proportions suitable for tyrosinase activity assays to analyze pH-dependent effects. Mushroom tyrosinase activity was measured across a range of pH values. For the anti-tyrosinase activity analysis, tyrosinase and L-DOPA were prepared using the following buffers: 100 mM glycine/HCl buffer (pH 2–3), 100 mM sodium acetate/acetic acid buffer (pH 4–5), and 100 mM sodium phosphate buffer (pH 6–8).

To investigate the influence of four carboxylic acids—3-phenyllactic acid, lactic acid, L-pyroglutamic acid, and malic acid—on pH in the absence of tyrosinase, and to assess their potential acidification effects on tyrosinase activity, pH measurements were conducted. Tyrosinase exhibited optimal activity at pH 6–7, with deviations from this range leading to reduced enzymatic activity (Figure 4). At a concentration of 3 mM, the pH values for 3-phenyllactic acid, lactic acid, and L-pyroglutamic acid ranged from 7.2 to 6.1, while malic acid significantly lowered the pH to 4.6. When the concentration was increased to 6 mM, 3-phenyllactic acid and lactic acid maintained a near-neutral pH of around 6. In contrast, L-pyroglutamic acid and malic acid further decreased the pH to 3.7 and 3.5, respectively. Lactic acid required a higher concentration of 12 mM to reach a pH of 3.6. These results indicate that malic acid has the strongest acidification effect, followed by L-pyroglutamic acid. Nevertheless, 3-phenyllactic acid and lactic acid maintain higher pH levels at lower concentrations.

**Figure 5:**
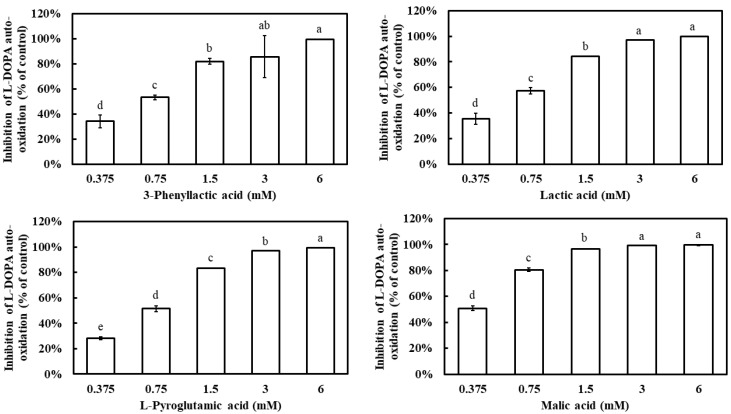
The inhibition of L-DOPA auto-oxidation was evaluated using varying concentrations of 3-phenyllactic acid, lactic acid, L-pyroglutamic acid, and malic acid. Distilled water was used as a control to assess the inhibitory effects of 3-phenyllactic acid, lactic acid, L-pyroglutamic acid, and malic acid treatments. The columns marked with different letters indicate statistically significant differences (*p* < 0.05). Data are expressed as mean ± standard deviation (S.D.) (*n* = 3).

Additionally, L-DOPA is known to undergo auto-oxidation in the absence of tyrosinase, leading to the formation of dopachrome. To evaluate whether the four carboxylic acids could inhibit L-DOPA auto-oxidation, their effects were examined. All four compounds demonstrated a dose-dependent inhibition of L-DOPA auto-oxidation, with inhibition rates significantly increasing as their concentrations increased (Figure 5). At 1.5 mM, 3-phenyllactic acid, lactic acid, and L-pyroglutamic acid achieved inhibition rates exceeding 80%, while malic acid reached the same inhibition rate at a lower concentration of 0.75 mM. The IC_50_ values for L-DOPA auto-oxidation inhibition, calculated using SPSS, were determined to be 0.63 mM for 3-phenyllactic acid, 0.57 mM for lactic acid, 0.66 mM for L-pyroglutamic acid, and 0.38 mM for malic acid.

### 2.4. Melanin Content Assay of B16-F10 Cells Treated by 3-Phenyllactic Acid, Lactic Acid, L-pyroglutamic Acid, and Malic Acid

**Figure 6:**
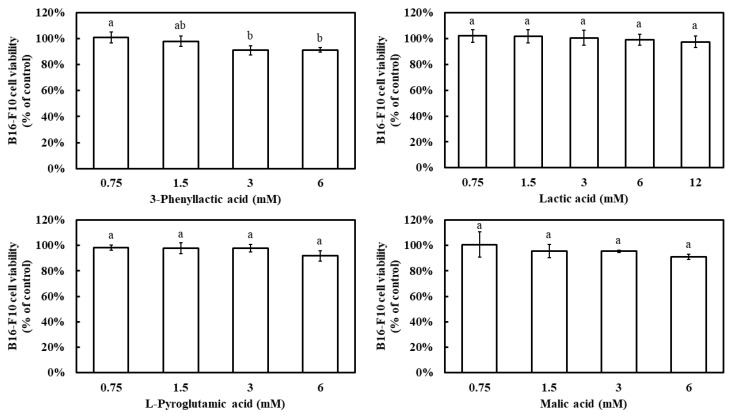
Effect of 3-phenyllactic acid, lactic acid, L-pyroglutamic acid, and malic acid on the viability of B16-F10 cells. Distilled water was used as a control to compare the impact of treatments with 3-phenyllactic acid, lactic acid, L-pyroglutamic acid, and malic acid. The columns marked with different letters indicate statistically significant differences (*p* < 0.05). Data are expressed as mean ± standard deviation (S.D.) (*n* = 3).

Prior to evaluating the inhibitory effects of the four carboxylic acids—3-phenyllactic acid, lactic acid, L-pyroglutamic acid, and malic acid—on melanin production in B16-F10 cells, their impact on cell viability was assessed using the MTT assay. The results demonstrated that, at a concentration of 6 mM, all four compounds maintained a B16-F10 cell viability above 90% (Figure 6). Notably, even at 12 mM, lactic acid exhibited a cell viability of 97%. These findings indicate that the concentration ranges used in this study did not significantly affect cell survival.

**Figure 7:**
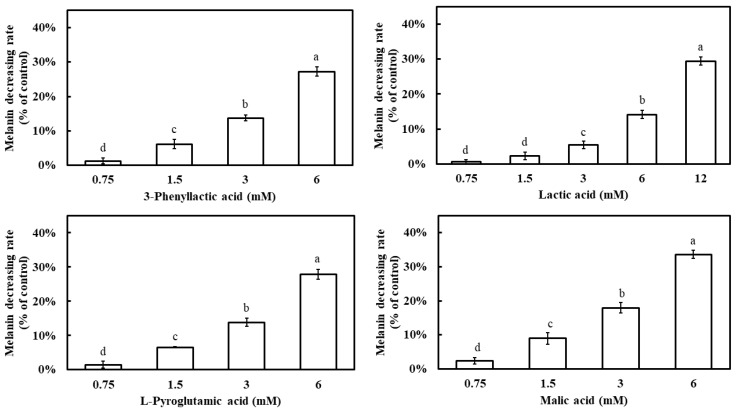
Effect of 3-phenyllactic acid, lactic acid, L-pyroglutamic acid, and malic acid on the melanin content of B16-F10 cells. Distilled water was used as a control to compare the impact of treatments with 3-phenyllactic acid, lactic acid, L-pyroglutamic acid, and malic acid. The columns marked with different letters indicate statistically significant differences (*p* < 0.05). Data are expressed as mean ± standard deviation (S.D.) (*n* = 3).

All four carboxylic acids exhibited a dose-dependent inhibition of melanin synthesis, with melanin inhibition rates increasing as their concentrations increased (Figure 7). At 6 mM, the melanin inhibition rates were 27.2% for 3-phenyllactic acid, 14.1% for lactic acid, 27.8% for L-pyroglutamic acid, and 33.6% for malic acid. When the concentration of lactic acid was increased to 12 mM, its melanin inhibition rate increased to 29.4%.

### 2.5. Proteomic Assay of B16-F10 Cells Treated by 3-Phenyllactic Acid, Lactic Acid, L-pyroglutamic Acid, and Malic Acid

To elucidate the mechanisms by which the four carboxylic acids—3-phenyllactic acid, lactic acid, L-pyroglutamic acid, and malic acid—inhibit melanin production in B16-F10 cells, a comparative proteomic analysis was conducted to assess changes in protein expression. Proteins with a fold change ≥ 2 were considered significantly regulated. The results revealed that 3-phenyllactic acid treatment led to the upregulation of 24 proteins and the downregulation of 83 proteins. Similarly, lactic acid induced the upregulation of 23 proteins and the downregulation of 61 proteins. L-Pyroglutamic acid resulted in the upregulation of 9 proteins and the downregulation of 32 proteins, while malic acid caused the upregulation of 14 proteins and the downregulation of 90 proteins. Detailed lists of the regulated proteins for each carboxylic acid are provided in Supplementary Materials Tables S1–S4.

**Figure 8:**
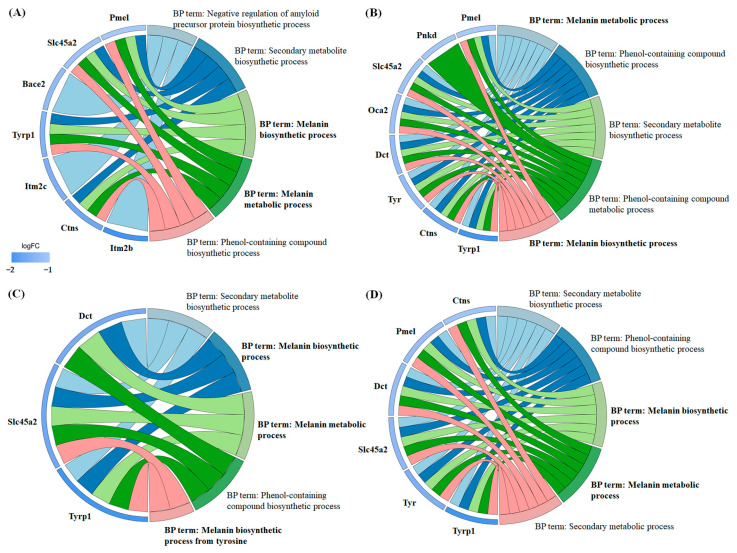
Chord diagrams were generated from GO enrichment analysis (Biological Process) to visualize the differentially expressed proteins in B16-F10 cells treated with four carboxylic acids: (**A**) 3-phenyllactic acid (6 mM), (**B**) lactic acid (12 mM), (**C**) L-pyroglutamic acid (6 mM), and (**D**) malic acid (6 mM).

Functional annotation of the differentially expressed proteins was performed using the Gene Ontology (GO) (Figure S1) and Kyoto Encyclopedia of Genes and Genomes (KEGG) (Figure S2) databases. The results demonstrated significant enrichment across multiple GO categories, including Biological Process (Figure S3), Molecular Function (Figure S4), and Cellular Component (Figure S5). A chord diagram was used to visualize the relationships between significantly enriched GO terms and the regulated proteins (Figure 8). Notably, in the Biological Process category, all four carboxylic acids exhibited enrichment in terms related to “melanin biosynthetic process” and “melanin metabolic process”, with the majority of the associated proteins showing downregulation. Specifically, in 3-phenyllactic-acid-treated cells, key genes involved in melanin synthesis and metabolism, such as Pmel, Slc45a2, Tyrp1, and Ctns, were downregulated. In lactic-acid-treated cells, the downregulated genes included Pmel, Slc45a2, Oca2, Dct, Tyr, Tyrp1, and Ctns. For L-pyroglutamic acid, the affected genes were Slc45a2, Dct, and Tyrp1. Finally, in malic-acid-treated cells, the downregulated genes encompassed Pmel, Slc45a2, Dct, Tyr, Tyrp1, and Ctns.

In the Cellular Component category (Figure S5), the differentially expressed proteins showed significant enrichment in melanosome membrane and pigment granule membrane-related terms across all four carboxylic acids. Moreover, 3-phenyllactic acid and malic acid were observed to potentially modulate vesicle membrane and lysosome functions. Lactic acid exhibited notable effects on cell-membrane-related components, including the cell surface, plasma membrane, and cell periphery. Conversely, L-pyroglutamic acid mainly influenced components linked to the cytoskeleton and neuronal structures, such as the axon membrane and synaptic membrane.

### 2.6. Network Toxicology Analysis of 3-Phenyllactic Acid, Lactic Acid, L-pyroglutamic Acid, and Malic Acid

**Figure 9:**
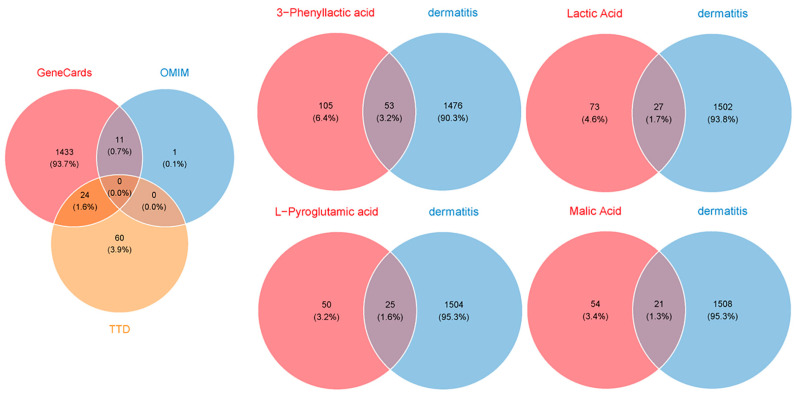
Venn diagrams were created to identify the overlapping target genes between dermatitis and the four carboxylic acid compounds: 3-phenyllactic acid, lactic acid, L-pyroglutamic acid, and malic acid. The target prediction for the four carboxylic acids was conducted using the SwissTargetPrediction, TargetNet, and SEA databases. The target genes associated with dermatitis were retrieved from the GeneCards, OMIM, and TTD databases.

To evaluate the potential of four carboxylic acids—3-phenyllactic acid, lactic acid, L-pyroglutamic acid, and malic acid—for skincare applications and their risk of inducing skin inflammation, a network toxicology approach was utilized to predict and analyze their interactions with dermatitis-related targets. Target prediction was performed using three online platforms: SwissTargetPrediction, TargetNet, and SEA. The predicted target genes for each compound were consolidated, yielding 158, 100, 75, and 75 targets for 3-phenyllactic acid, lactic acid, L-pyroglutamic acid, and malic acid, respectively (Figure 9). Furthermore, 1529 dermatitis-related genes were identified from the GeneCards, OMIM, and TTD databases.

**Figure 10:**
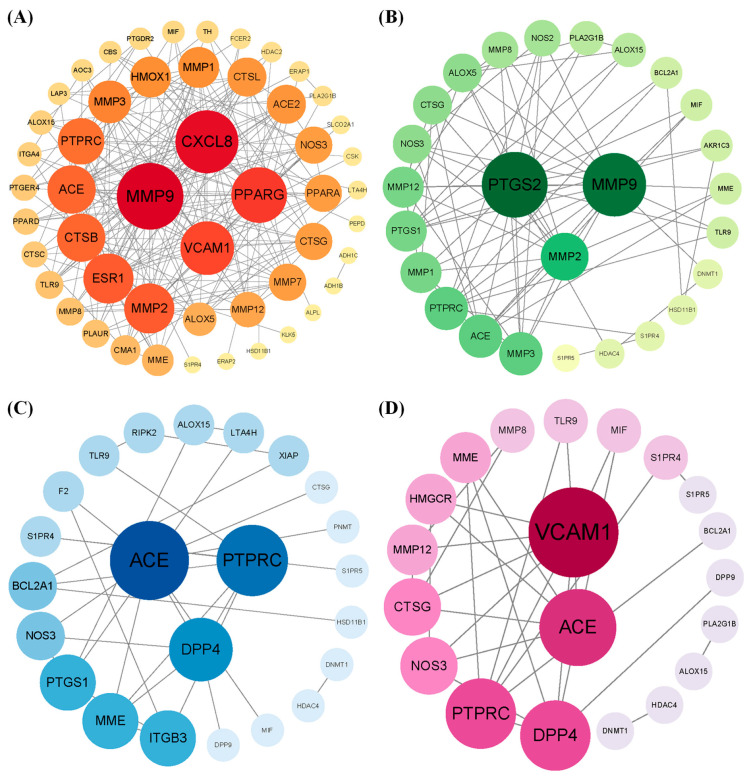
The protein–protein interaction (PPI) network of core targets associated with (**A**) 3-phenyllactic acid, (**B**) lactic acid, (**C**) L-pyroglutamic acid, and (**D**) malic acid was constructed and visualized using Cytoscape.

By intersecting the predicted targets with dermatitis-related genes, we identified 53, 27, 25, and 21 shared targets for 3-phenyllactic acid, lactic acid, L-pyroglutamic acid, and malic acid, respectively. These shared targets were subsequently analyzed through GO (Figure S6) and KEGG pathway enrichment analyses (Figure S7) to uncover their functional roles. The targets were then imported into the STRING database to construct a protein–protein interaction (PPI) network, with the biological species specified as *Homo sapiens*. The PPI network was visualized and analyzed using Cytoscape v3.10.0 to identify core dermatitis-related targets (Figure 10). The core targets for 3-phenyllactic acid included MMP9, CXCL8, PPARG, and VCAM1. For lactic acid, the core targets were MMP2, PTGS2, and MMP9. L-Pyroglutamic acid exhibited core targets such as ACE, DPP4, and PTPRC, while malic acid targeted ACE and VCAM1. Molecular docking was performed to assess the binding affinity between these core targets and their respective compounds. The results, presented in Table 1, revealed that only 3-phenyllactic acid demonstrated strong binding stability with MMP9 and PPARG, as evidenced by binding energies below -6 kcal/mol. Although the other carboxylic acids exhibited higher binding energies, they still displayed potential binding capabilities through hydrogen bonds (H-bonds), weak hydrogen bonds (Weak H-bonds), and ionic interactions.

## 3. Discussion

Four carboxylic acid compounds—3-phenyllactic acid, lactic acid, L-pyroglutamic acid, and malic acid—demonstrated notable tyrosinase inhibitory activity (Figure 1). This effect aligns with other known carboxylic acid inhibitors including kojic acid, azelaic acid, ascorbic acid, lipoic acid, fumaric acid, α/β-hydroxy acids, 2-chlorocinnamic acid, and 2,4-dichlorocinnamic acid [23,24,25]. The inhibitory potency of these carboxylic acid compounds varies significantly depending on the source of tyrosinase. For instance, kojic acid, a mixed-type tyrosinase inhibitor, exhibits an IC_50_ value greater than 0.5 mM for human tyrosinase [26]. However, Ismail et al. reported a markedly lower IC_50_ value of 9.27 µM for kojic acid using tyrosinase from BioVision, Inc. (claimed to be of human origin) [24]. Similarly, their study on malic acid revealed an IC50 value of 87.46 µM, whereas our analysis using mushroom tyrosinase yielded a significantly higher IC_50_ value of 3.91 mM. In our previous studies, succinic acid and citric acid exhibited IC_50_ values of 2.943 mM and 1.615 mM, respectively, for mushroom tyrosinase inhibition [21]. Meanwhile, 2-chlorocinnamic acid and 2,4-dichlorocinnamic acid showed IC_50_ values of 0.765 mM and 0.295 mM, respectively, in the same mushroom tyrosinase assay [25]. These findings suggest that carboxylic acid compounds generally tend to exhibit higher IC_50_ values when tested with mushroom tyrosinase compared to other sources. This variability underscores the importance of considering the enzyme source when evaluating tyrosinase inhibition efficacy.

Furthermore, the inhibitory effects of these compounds on tyrosinase may not be direct, but rather mediated through environmental acidification. Previous research has demonstrated that an acidic microenvironment can substantially suppress melanin synthesis by inhibiting tyrosinase activity [21,27]. This acidification mechanism is crucial for regulating melanosome function and provides insight into the pathological basis of certain albinism types. Among the four carboxylic acid compounds examined, malic acid exhibited the strongest acidifying capacity, followed by L-pyroglutamic acid (Figure 4). Analysis of their tyrosinase inhibition rates suggests that malic acid primarily exerts its inhibitory effect through environmental acidification, which subsequently reduces enzyme activity. In contrast, L-pyroglutamic acid and lactic acid appear to inhibit tyrosinase through a dual mechanism: environmental acidification combined with intrinsic tyrosinase-inhibitory properties. This acidification-mediated suppression of tyrosinase activity has been previously documented with other compounds, notably ascorbic acid and its derivatives, which achieve tyrosinase inhibition through cytoplasmic acidification [28]. Additionally, acidic conditions have been shown to enhance the binding affinity of salicylic acid to tyrosinase by promoting hydrogen bonding and electrostatic interactions [29]. Similar to salicylic acid, L-pyroglutamic acid and lactic acid likely inhibit tyrosinase through a combination of acidification effects and direct binding interactions, representing a more complex inhibitory mechanism than that of malic acid.

In contrast to the other compounds, 3-phenyllactic acid demonstrated a unique inhibition profile. Within the tested concentration range (0.75 mM to 6 mM), its pH remained between 6 and 7, coinciding with the optimal pH range for tyrosinase activity. This observation strongly suggests that 3-phenyllactic acid’s inhibitory effect was primarily mediated through its intrinsic molecular properties rather than environmental acidification. This hypothesis was further substantiated by comprehensive molecular docking analyses of the interactions between the four compounds and both mouse and mushroom tyrosinase. Notably, among all carboxylic acids, 3-phenyllactic acid displayed the most favorable binding energetics with tyrosinase (Table S5). Specifically, it exhibited a binding energy of −6.2 kcal/mol with mouse tyrosinase, stabilized by the formation of six hydrogen bonds. Even more remarkably, its interaction with mushroom tyrosinase showed enhanced binding affinity, with a lower binding energy of −6.6 kcal/mol supported by five conventional hydrogen bonds and one weak hydrogen bond. These findings not only corroborate the initial hypothesis but also provide molecular-level insights into the distinctive inhibition mechanism of 3-phenyllactic acid, setting it apart from the other tested compounds.

The inhibition of melanogenesis is not solely limited to the suppression of tyrosinase activity. Another critical approach in screening melanin inhibitors involves suppressing the oxidation of DOPA to dopaquinone, thereby preventing the formation of dopachrome [11]. For instance, compounds such as baicalein, rifampicin, and ascorbic acid act as dopaquinone reductants, effectively inhibiting the production of dopachrome and melanin [12,13,14]. Additionally, thiol compounds are significant inhibitors that react with dopaquinone to form colorless products, thereby substantially reducing melanin synthesis [15]. In the process of screening tyrosinase inhibitors, some studies have revealed that certain compounds not only inhibit tyrosinase activity but also inhibit DOPA auto-oxidation. For example, *Ginkgo biloba* L. extract exhibits an IC_50_ value of 211.9 µg/mL for tyrosinase inhibition and an IC_50_ value of 456.3 µg/mL for DOPA auto-oxidation inhibition [30]. Comparative analysis shows that kojic acid and ascorbic acid exhibit IC_50_ values of 418.5 µg/mL and 989.6 µg/mL, respectively, for DOPA auto-oxidation inhibition. Similarly, *Alnus cordata* (Loisel.) Duby extract demonstrates potent tyrosinase inhibition (IC_50_ = 77.4 µg/mL) alongside significant suppression of DOPA auto-oxidation (IC_50_ = 39.6 µg/mL) [31]. Pistachio hull extract also demonstrates this dual functionality, exhibiting an IC_50_ of 116.1 µg/mL for mushroom tyrosinase inhibition and significant DOPA auto-oxidation suppression within the concentration range of 125–500 µg/mL [32]. In this study, all four carboxylic acid compounds demonstrated inhibitory effects on DOPA auto-oxidation, with their IC_50_ values for DOPA auto-oxidation inhibition being lower than those for tyrosinase inhibition (Figure 5). Among them, malic acid showed the lowest IC_50_ value (0.38 mM), followed by lactic acid (0.57 mM), 3-phenyllactic acid (0.63 mM), and L-pyroglutamic acid (0.66 mM). These results suggest that the anti-melanogenic effects of these carboxylic acids were mediated through a multifaceted mechanism involving (1) direct inhibition of tyrosinase catalytic activity, (2) microenvironment acidification, and (3) suppression of DOPA auto-oxidation pathways.

Through comprehensive proteomic analysis, this study systematically investigated the impact of four carboxylic acid compounds on B16-F10 cells, revealing significant alterations in melanogenesis-related protein expression. The results revealed that all four compounds downregulated tyrosinase-related protein 1 (Tyrp1), a key protein directly involved in melanin synthesis (Figure 8). Additionally, lactic acid, L-pyroglutamic acid, and malic acid were found to suppress the expression of tyrosinase-related protein 2 (Tyrp2, also known as dopachrome tautomerase, Dct). Nevertheless, only lactic acid and malic acid reduced the expression of tyrosinase. Notably, the expression of MITF, a critical transcription factor of melanogenic proteins, remained largely unaffected. While a previous study by Usuki et al. demonstrated no significant regulatory effects of lactic acid on melanogenesis-related proteins (tyrosinase and Tyrp 1/2) through Western blot analysis [33], our current findings presented a contrasting perspective. This apparent discrepancy may be attributed to fundamental differences in experimental design, particularly regarding lactic acid concentration and treatment regimen. Usuki et al. utilized comparatively lower lactic acid concentrations (300 and 500 μg/mL, corresponding to ~3.33 and 5.55 mM, respectively) administered intermittently (48 h intervals during a 5 d culture period). These experimental conditions may have limited lactic acid’s ability to modulate melanogenic protein expression. Furthermore, in our study, other proteins associated with melanin synthesis and metabolism also exhibited downregulation, including Pmel, Slc45a2, Ctns, and Oca2. Pmel (Premelanosome protein) is a pigment-cell-specific protein primarily responsible for forming fibrillar sheets within melanosomes, which serve as templates for melanin polymerization and deposition [34,35]. Interestingly, Pmel expression is not restricted to pigment cells; it has also been detected in non-pigmented tissues of cattle, such as the thyroid, liver, and kidneys. This widespread distribution suggests potential functions beyond melanin deposition, such as modulating pheomelanin dilution or influencing hair-related phenotypes [34]. However, the precise roles of Pmel in non-pigmented tissues remain to be elucidated.

The Slc45a2 gene plays a pivotal role in melanin synthesis and pigmentation across various species, including chickens, mice, and parrots [36,37,38]. In mice, Slc45a2 functions as a proton/glucose exporter in melanosomes, maintaining melanosomal pH and regulating tyrosinase activity [36]. Its deficiency leads to melanosome acidification, impaired melanin synthesis, and enhanced glycolysis, thereby promoting melanoma metastasis. In parrots, mutations in Slc45a2 disrupt melanin production, resulting in altered feather coloration [37]. The Ctns gene encodes cystinosin, a cystine/H^+^ transporter crucial for cysteine homeostasis and melanogenesis [39,40]. Mutations in Ctns cause cystinosis, a rare autosomal recessive disorder characterized by lysosomal cystine accumulation, and pigmentation abnormalities such as blond hair and fair skin [40]. Studies on cystinosis patients and Ctns-deficient mice have demonstrated a significant reduction in eumelanin (black/brown pigment) and an increase in pheomelanin (yellow/red pigment), indicating disrupted melanin synthesis due to impaired tyrosinase activity and lysosomal protease-mediated degradation. Oculocutaneous albinism (OCA) is a genetic disorder caused by mutations in genes involved in melanin synthesis, including Tyr (OCA-1), Oca2 (OCA-2), Tyrp1 (OCA-3), and Slc45a2 (OCA-4) [41]. The functional role of Oca2 in melanin synthesis has been further elucidated through CRISPR/Cas9 gene editing in *Astatotilapia callipera* [42].

Although Bace2 (β-site amyloid precursor protein-cleaving enzyme 2) is primarily recognized as a negative regulator of amyloid precursor protein biosynthesis (Figure 8), emerging evidence has revealed its significant role in melanogenesis. Bace2 facilitates the formation of amyloid fibrils in melanosomes by cleaving Pmel within its juxtamembrane domain [43]. Inhibition of Bace2, whether through genetic manipulation or pharmacological intervention (using dual Bace1/Bace2 inhibitors), disrupts Pmel processing, leading to impaired melanin storage and hair depigmentation in mice [44]. While Bace1 is mainly associated with amyloid-β (Aβ) production in Alzheimer’s disease, its structural homology to Bace2 suggests that its inhibition may also impact melanin synthesis [44]. In summary, the effects of these four carboxylic acid compounds on intracellular melanin extend beyond proteins directly involved in melanin synthesis to include other proteins regulating melanin metabolism. This may be attributed to their ability to alter intracellular pH or reduce DOPA and other intermediates, thereby indirectly downregulating melanin-related proteins. Therefore, the regulatory effects of these compounds on Pmel, Slc45a2, Ctns, Oca2, and Bace2 represent a promising area for further in-depth investigation.

To assess the suitability of these four carboxylic acid compounds for human skin and their potential to induce skin inflammation, we utilized network toxicology methods to predict their potential targets related to human dermatitis (Figure 9). According to the Flavor and Extract Manufacturers Association of the United States (FEMA), lactic acid (FEMA No. 2611) and malic acid (FEMA No. 2655) are classified as “Generally Recognized as Safe” (GRAS) substances. Previous studies have further supported their safety profile by reporting high median lethal dose (LD_50_) values for these compounds. Specifically, the oral LD_50_ values for lactic acid in rats, mice, and guinea pigs are 3730 mg/kg, 4875 mg/kg, and 1810 mg/kg, respectively, while the oral LD_50_ values for malic acid in rabbits and mice range from 1600 mg/kg to 3200 mg/kg [45,46]. However, toxicological data for 3-phenyllactic acid and L-pyroglutamic acid are currently limited. Predictions from the ProTox 3.0 database estimate their LD_50_ values in rodents to be 1154 mg/kg and 1000 mg/kg, respectively [47]. Both compounds are classified as Class IV (“Harmful if swallowed”) in the database’s six-tier toxicity classification system.

Using network toxicology, we analyzed the four carboxylic acid compounds and found that, except for 3-phenyllactic acid, which exhibited favorable binding energies to matrix metalloproteinase-9 (MMP9) and peroxisome proliferator-activated receptor gamma (PPARG or PPARγ), the binding energies of the other compounds were above -6.0 kcal/mol (Table 1). MMP9, also known as gelatinase B, is a protease capable of degrading type IV collagen in the extracellular matrix and basement membrane, playing a significant role in inflammatory responses and tissue destruction [48]. During inflammation, the expression and activity of MMP9 are markedly increased. Therefore, if 3-phenyllactic acid can effectively inhibit MMP9 activity, it may potentially exert a positive effect on suppressing skin inflammation. PPARγ, a nuclear receptor, is known to reduce inflammation and oxidative stress, promote cell cycle arrest, and induce apoptosis, offering new avenues for cancer treatment [49]. However, based on our proteomic analysis, 3-phenyllactic acid did not significantly downregulate MMP9 or PPARG in mouse B16-F10 cells. In summary, these carboxylic acid compounds exhibited a relatively low potential risk of inducing skin inflammation and hold promise for future applications in inhibiting melanogenesis.

## 4. Materials and Methods

### 4.1. Inhibition of Tyrosinase Activity

L-DOPA solution (5 mM) and tyrosinase (400 U/mL) (Sigma-Aldrich, St. Louis, MO, USA) were prepared in PBS buffer (pH 7.4) (Qingdao Hope Bio-Technology Co., Ltd., Qingdao, China). A reaction mixture was prepared by combining 240 μL of carboxylic acid at various concentrations, 360 μL of L-DOPA solution, and 120 μL of tyrosinase. The mixture was incubated in the dark for 5 min, after which the absorbance was measured at 475 nm using a microplate reader (Infinite 200Pro, Tecan, Männedorf, Switzerland). Deionized water was used as the control. The inhibition rate of tyrosinase was calculated with Equation (1):

(1) [(OD475 of control−OD475 of sample)/OD475 of control]×100%

### 4.2. Fluorescence Spectra Analysis of Tyrosinase

Carboxylic acid solutions at various concentrations were mixed with PBS buffer and tyrosinase (final concentration of 60 U/mL) and then transferred to a black 96-well plate. Fluorescence analysis was conducted using a microplate reader (Infinite 200Pro, Tecan). The instrument was configured for automated quantitative plotting with the following parameters: Fluorescence Intensity Scan; excitation wavelength fixed at 280 nm; emission wavelength range of 305 to 500 nm; and a step size of 1 nm.

### 4.3. Kinetic Analysis of Tyrosinase

L-DOPA solutions at various concentrations (0.5, 1, 2, and 4 mM) were prepared in PBS buffer (pH 7.4) and mixed with carboxylic acids at different concentrations and tyrosinase (final concentration of 60 U/mL). The reaction mixture was incubated in the dark for 5 min, after which the absorbance at 475 nm was immediately measured using a microplate reader (Infinite 200Pro, Tecan). The reaction rates were calculated based on the OD values. To analyze the inhibition types of the four carboxylic acids on tyrosinase, double-reciprocal Lineweaver–Burk plots were constructed with 1/v (reaction rate) as the *y*-axis and 1/[S] (substrate concentration) as the *x*-axis. The maximum reaction velocity (V_max_) and Michaelis constant (K_m_) were determined using GraphPad Prism 10 (GraphPad Software, Massachusetts, USA). The inhibition constants (K_i_) were calculated using both mixed-model inhibition and competitive inhibition models.

### 4.4. Cell Viability of B16-F10 Measured by MTT Method

B16-F10 cells were seeded in 96-well plates at a density of 1 × 10^5^ cells/mL and cultured at 37 °C with 5% CO2 for 24 h. Subsequently, various concentrations of carboxylic acids were added, and the cells were further incubated for 24 h. Then, 6 μL of MTT (5 mg/mL) was added, and the cells were cultured for an additional 4 h. Afterward, 200 μL of DMSO was added to dissolve the formazan crystals by gently pipetting up and down. The absorbance at 570 nm (OD570) was measured using a microplate reader (Infinite 200Pro, Tecan), with deionized water serving as the control. The cell viability was calculated with Equation (2):

(2) [(OD570 of sample)/OD570 of control]×100%

### 4.5. Melanin Content Analysis of B16-F10 Cells

A 900 μL cell suspension with an approximate density of 50% confluence was seeded into a 12-well plate and cultured for 24 h at 37 °C with 5% CO_2_. Subsequently, various concentrations of carboxylic acids were added, and the cells were further incubated for 1 h. Then, 1 μL of 1 mM α-melanocyte-stimulating hormone (α-MSH) solution was added to achieve a final concentration of 1 μM, and the cells were cultured for an additional 48 h at 37 °C with 5% CO_2_ to stimulate melanogenesis. Afterward, the cells were digested with trypsin, collected by centrifugation at 10,000 rpm for 5 min (Andreas Hettich GmbH & Co., KG, Tuttlingen, Germany), and the supernatant was discarded. Each tube was treated with 200 μL of 1 M NaOH solution and incubated in a water bath at 60 °C for 20 min. Following incubation, the samples were vortexed to ensure homogeneity, and 200 μL of each sample was transferred to a 96-well plate. The absorbance at 405 nm (OD405) was measured using a microplate reader (Infinite 200Pro, Tecan), and the data were recorded to calculate the melanin content, with deionized water serving as the control. The reduction in melanin content was calculated with Equation (3):

(3) [(OD405 of control−OD405 of sample)/OD405 of control]×100%

### 4.6. L-DOPA Auto-Oxidation Analysis

A 5 mM L-DOPA solution was prepared in PBS buffer (pH 7.4). Subsequently, 240 μL of carboxylic acid solution, 360 μL of L-DOPA solution, and 120 μL of PBS buffer were sequentially mixed. The mixture was then incubated at 37 °C for 12 h. After incubation, the absorbance at 475 nm was measured, and the data were recorded. Deionized water was used as the control. The inhibition of L-DOPA auto-oxidation was calculated using the same method as the melanin content analysis.

### 4.7. Proteomic Analysis

B16-F10 cells were treated with various concentrations of carboxylic acids following the same culture protocol used for melanin inhibition assays. After treatment, the cells were collected and submitted to Shanghai Zhongke Xinming Biotechnology Co., Ltd. (Shanghai, China) for proteomics analysis. The collected cells were lysed with an appropriate volume of SDT lysis buffer (4% SDS, 100 mM Tris-HCl, pH 7.6) to extract proteins. DTT was added to reduce disulfide bonds, followed by incubation at room temperature for 1 h. Subsequently, iodoacetamide was added, and the mixture was reacted in the dark at room temperature for 30 min. The proteins were then digested with trypsin overnight. The resulting peptide fragments were desalted using a C18 cartridge. After freeze-drying, the peptides were resuspended in 40 μL of 0.1% formic acid solution, and the peptide concentration was determined by measuring the absorbance at 280 nm. An appropriate amount of internal reference for targeted proteomics (iRT) standard peptides was added to each sample for data-independent acquisition (DIA) mass spectrometry analysis. Chromatographic separation was performed using a nanoflow Evosep One system. The separated samples were analyzed by DIA mass spectrometry using a timsTOF Pro mass spectrometer (Bruker Daltonics, Bremen, Germany). Detection was conducted in positive-ion mode, with MS and MS/MS scanning ranges set from 100 to 1700 *m*/*z*. MS2 data acquisition was performed in DIA mode, with four TIMS scan windows, each accumulating for 100 ms. In PASEF mode, the collision energy varied linearly with ion mobility (1/K0), ranging from 20 to 59 eV for ions with 1/K0 values between 0.85 and 1.30 Vs/cm^2^. The DIA data were processed using Spectronaut 19 software with the following parameter settings: retention time prediction type set to dynamic iRT, interference correction on MS2 level enabled, cross-run normalization enabled, maximum missed cleavage sites set to 2, fixed modification set to Carbamidomethyl (C), and variable modifications set to Oxidation (M) and Acetyl (Protein N-term). All results were filtered with a Q-value cutoff of 0.01, corresponding to a false discovery rate (FDR) of less than 1%. The target protein set was annotated using Blast2GO (version BLASTP 2.8.0+). The process included four steps: sequence alignment (Blast), GO mapping, GO annotation, and additional annotation augmentation using InterProScan [50]. Using the KOBAS 3.0 software, KEGG pathway annotation was performed on the target protein set [51]. Fisher’s exact test was employed to compare the distribution of GO categories in the target protein set and the overall protein set, thereby conducting enrichment analysis of the GO annotations in the target protein set. The proteomics dataset generated in this study has been made publicly available through the PRIDE ProteomeXchange Consortium [52] with the dataset identifier PXD061919.

### 4.8. **Network** Toxicology Analysis

The network toxicology workflow began with toxicity analysis of the compounds using ProTox 3.0 [47], followed by compound target prediction through SwissTargetPrediction [53], TargetNet [54], and SEA Search Server [55], with the results combined by taking their union. Subsequently, relevant disease names (e.g., dermatitis) were identified, and disease-related targets were retrieved from GeneCards [56], OMIM [57], and TTD [58], with the results also combined by taking their union. The intersection of compound target genes and disease-related genes was determined using R 4.4.1. A compound regulatory network was constructed using Cytoscape [59], followed by the generation of a protein–protein interaction network using the STRING database. Core genes in the network were identified using Cytoscape. Finally, the protein structures of the core genes were obtained, and molecular docking was performed using CB-Dock2 [60].

## 5. Conclusions

This study comprehensively elucidated the multiple mechanisms and potential applications of four carboxylic acid compounds—3-phenyllactic acid, lactic acid, L-pyroglutamic acid, and malic acid—in inhibiting melanogenesis. The results demonstrated that these compounds reduce melanin synthesis through multiple pathways, including direct inhibition of tyrosinase activity, acidification of the microenvironment, and suppression of DOPA auto-oxidation. Among these compounds, malic acid primarily exerts its effects through environmental acidification to reduce tyrosinase activity, whereas 3-phenyllactic acid mainly relies on its intrinsic inhibitory properties. Furthermore, all four compounds significantly inhibit DOPA auto-oxidation, underscoring their multi-mechanistic roles in regulating melanogenesis. Proteomic analysis revealed the broad regulatory effects of these compounds on key proteins involved in melanin synthesis, including the downregulation of Tyrp1, Tyrp2, Pmel, Slc45a2, Ctns, and Oca2. Notably, the critical roles of Pmel and Slc45a2 in melanosome formation and function, combined with the regulatory functions of Ctns and Oca2 in melanin metabolism, further highlight the complex network through which these compounds modulate melanogenesis. Additionally, the potential involvement of Bace2 suggests that these compounds might indirectly influence melanin storage by regulating amyloid fibril formation. Network toxicology analysis indicated a low risk of these carboxylic acid compounds inducing skin inflammation, with certain compounds (e.g., 3-phenyllactic acid) potentially exerting positive effects on MMP9 and PPARγ. Given their high safety profiles and low toxicity, these compounds exhibit significant promise for future applications in melanogenesis inhibition.

## Figures and Tables

**Table 1:** Molecular docking analysis was performed using CB-Dock2 to evaluate the binding interactions between the core targets identified from the PPI network and the following carboxylic acids: 3-phenyllactic acid, lactic acid, L-pyroglutamic acid, and malic acid.

Compounds	Enzymes	Binding Energy (kcal/mol)	Pi–Pi	Pi–Cation	H-Bond	Weak H-Bond	Ionic Interaction
3-Phenyllactic acid	MMP9	−7.8	1	0	4	1	2
	CXCL8	−5	1	1	1	0	0
	PPARG	−6.5	0	0	3	0	1
	VCAM1	−5.8	0	0	6	0	1
Lactic acid	MMP9	−4.3	0	0	6	1	1
	MMP2	−4.2	0	0	5	0	0
	PTGS2	−4.6	0	0	2	0	1
L-Pyroglutamic acid	ACE	−5.2	0	0	3	1	0
	DPP4	−5.3	0	0	3	0	1
	PTPRC	−5.3	0	0	5	0	0
Malic acid	VCAM1	−4.8	0	0	2	0	0
	ACE	−5.3	0	0	5	0	0

## Data Availability

The raw data supporting the conclusions of this article will be made available by the authors on request.

## Supplementary Materials

The following supporting information can be downloaded at https://www.mdpi.com/article/10.3390/molecules30071642/s1: Figure S1: GO analysis was performed on differentially expressed proteins in B16-F10 cells treated with (A) 3-phenyllactic acid (6 mM), (B) lactic acid (12 mM), (C) L-pyroglutamic acid (6 mM), and (D) malic acid (6 mM); Figure S2: KEGG analysis was performed on differentially expressed proteins in B16-F10 cells treated with (A) 3-phenyllactic acid (6 mM), (B) lactic acid (12 mM), (C) L-pyroglutamic acid (6 mM), and (D) malic acid (6 mM); Figure S3: GO enrichment analysis (Biological Process with top 20) was performed on differentially expressed proteins in B16-F10 cells treated with (A) 3-phenyllactic acid (6 mM), (B) lactic acid (12 mM), (C) L-pyroglutamic acid (6 mM), and (D) malic acid (6 mM); Figure S4: GO enrichment analysis (Molecular Function with top 20) was performed on differentially expressed proteins in B16-F10 cells treated with (A) 3-phenyllactic acid (6 mM), (B) lactic acid (12 mM), (C) L-pyroglutamic acid (6 mM), and (D) malic acid (6 mM); Figure S5: GO enrichment analysis (Cellular Component with top 20) was performed on differentially expressed proteins in B16-F10 cells treated with (A) 3-phenyllactic acid (6 mM), (B) lactic acid (12 mM), (C) L-pyroglutamic acid (6 mM), and (D) malic acid (6 mM); Figure S6: GO enrichment analysis was conducted to reveal the core targets associated with dermatitis-related genes and (A) 3-phenyllactic acid, (B) lactic acid, (C) L-pyroglutamic acid, and (D) malic acid; Figure S7: Enrichment analysis of KEGG pathway was conducted to reveal the core targets associated with dermatitis-related genes and (A) 3-phenyllactic acid, (B) lactic acid, (C) L-pyroglutamic acid, and (D) malic acid; Table S1: Differentially expressed proteins, including both upregulated and downregulated proteins, were identified in B16-F10 cells by comparing the 3-phenyllactic acid treatment group to the control group; Table S2: Differentially expressed proteins, including both upregulated and downregulated proteins, were identified in B16-F10 cells by comparing the lactic acid treatment group to the control group; Table S3: Differentially expressed proteins, including both upregulated and downregulated proteins, were identified in B16-F10 cells by comparing the L-pyroglutamic acid treatment group to the control group; Table S4: Differentially expressed proteins, including both upregulated and downregulated proteins, were identified in B16-F10 cells by comparing the malic acid treatment group to the control group; Table S5: Molecular docking analysis was performed using CB-Dock2 to evaluate the binding interactions between the tyrosinases and the following carboxylic acids: 3-phenyllactic acid, lactic acid, L-pyroglutamic acid, and malic acid.

## Abbreviations

The following abbreviations are used in this manuscript:

MITF	Microphthalmia-associated transcription factor
GO	Gene Ontology
KEGG	Kyoto Encyclopedia of Genes and Genomes
Tyrp1	Tyrosinase-related protein 1
Tyrp2	Tyrosinase-related protein 2
Dct	Dopachrome tautomerase
Pmel	Premelanosome protein
Slc45a2	Solute carrier family 45 member 2
Ctns	Cystinosin
OCA	Oculocutaneous albinism
Bace2	β-Site amyloid precursor protein-cleaving enzyme 2
MMP9	Matrix metalloproteinase-9
PPARγ	Peroxisome proliferator-activated receptor gamma

## References

[1] Tan M.G., Kim W.B., Jo C.E., Nabieva K., Kirshen C., Ortiz A.E. (2022). Topical treatment for postinflammatory hyperpigmentation: A systematic review. J. Dermatolog. Treat..

[2] Fatima S., Braunberger T., Mohammad T.F., Kohli I., Hamzavi I.H. (2020). The role of sunscreen in melasma and postinflammatory hyperpigmentation. Indian J. Dermatol..

[3] Tisack A., Mohammad T.F. (2024). Drug-Induced Pigmentation: A Review. Drugs.

[4] Mobasher P., Foulad D.P., Raffi J., Zachary C., Fackler N., Zohuri N., Juhasz M., Atanaskova Mesinkovska N. (2020). Catamenial hyperpigmentation: A review. J. Clin. Aesthet. Dermatol..

[5] Patil S.S., Patil S.M., Campbell R., Singh M., Plotkin M. (2020). Levofloxacin-induced acute hyperpigmentation changes in a chronic kidney disease patient. Case Rep. Med..

[6] Lee K.P., Vangipuram R.K., Klimas N.K., Sanyal S., Koshelev M.V. (2019). Hydroxyurea-induced hyperpigmentation with iron deposition. Dermatol. Online J..

[7] Zhu Y., Zeng X., Ying J., Cai Y., Qiu Y., Xiang W. (2022). Evaluating the quality of life among melasma patients using the MELASQoL scale: A systematic review and meta-analysis. PLoS ONE.

[8] Gunia-Krzyżak A., Popiol J., Marona H. (2016). Melanogenesis inhibitors: Strategies for searching for and evaluation of active compounds. Curr. Med. Chem..

[9] Hsiao J.J., Fisher D.E. (2014). The roles of microphthalmia-associated transcription factor and pigmentation in melanoma. Arch. Biochem. Biophys..

[10] Mo X., Preston S., Zaidi M.R. (2019). Macroenvironment-gene-microenvironment interactions in ultraviolet radiation-induced melanomagenesis. Adv. Cancer Res..

[11] Chang T.S. (2009). An updated review of tyrosinase inhibitors. Int. J. Mol. Sci..

[12] Gąsowska-Bajger B., Wojtasek H. (2023). Oxidation of baicalein by tyrosinase and by o-quinones. Int. J. Biol. Macromol..

[13] Tarasek D., Wojtasek H. (2022). Rifampicin is not an inhibitor of tyrosinase. Int. J. Biol. Macromol..

[14] Tomita Y., Hariu A., Mizuno C., Seiji M. (1980). Inactivation of tyrosinase by dopa. J. Investig. Dermatol..

[15] Boo Y.C. (2022). Metabolic basis and clinical evidence for skin lightening effects of thiol compounds. Antioxidants.

[16] Crous C., Swart I.A., Pretorius J., van der Kooy F., Petzer J.P., Petzer A. (2024). Hematoxylin, an alternative substrate of tyrosinase. Planta Med..

[17] Ortiz-Ruiz C.V., Ballesta de Los Santos M., Berna J., Fenoll J., Garcia-Ruiz P.A., Tudela J., Garcia-Canovas F. (2015). Kinetic characterization of oxyresveratrol as a tyrosinase substrate. IUBMB Life.

[18] Garcia-Jimenez A., Teruel-Puche J.A., Garcia-Ruiz P.A., Saura-Sanmartin A., Berna J., Rodríguez-López J.N., Garcia-Canovas F. (2018). Action of tyrosinase on caffeic acid and its n-nonyl ester. Catalysis and suicide inactivation. Int. J. Biol. Macromol..

[19] Zolghadri S., Bahrami A., Hassan Khan M.T., Munoz-Munoz J., Garcia-Molina F., Garcia-Canovas F., Saboury A.A. (2019). A comprehensive review on tyrosinase inhibitors. J. Enzyme Inhib. Med. Chem..

[20] Chen M.Y., Wu H.T., Chen F.F., Wang Y.T., Chou D.L., Wang G.H., Chen Y.P. (2022). Characterization of Tibetan kefir grain-fermented milk whey and its suppression of melanin synthesis. J. Biosci. Bioeng..

[21] Zhong L., He Q., Xu M., Chen F.-F., Li F., Chen Y.-P. (2024). Unveiling *Acetobacter syzygii* from Tibetan kefir grain: Fermentation-enhanced anti-tyrosinase, and anti-melanin. Fermentation.

[22] Shin M., Truong V.L., Lee M., Kim D., Kim M.S., Cho H., Jung Y.H., Yang J., Jeong W.S., Kim Y. (2023). Investigation of phenyllactic acid as a potent tyrosinase inhibitor produced by probiotics. Curr. Res. Food Sci..

[23] Zaid A.N., Al Ramahi R. (2019). Depigmentation and anti-aging treatment by natural molecules. Curr. Pharm. Des..

[24] Ismail M., Hassan M.H.A., Mohamed E.I.A., Azmy A.F., Moawad A., Mohammed R., Zaki M.A. (2024). New insights into the anti-inflammatory and anti-melanoma mechanisms of action of azelaic acid and other *Fusarium solani* metabolites via in vitro and in silico studies. Sci. Rep..

[25] Hu Y.H., Liu X., Jia Y.L., Guo Y.J., Wang Q., Chen Q.X. (2014). Inhibitory kinetics of chlorocinnamic acids on mushroom tyrosinase. J. Biosci. Bioeng..

[26] Mann T., Gerwat W., Batzer J., Eggers K., Scherner C., Wenck H., Stäb F., Hearing V.J., Röhm K.H., Kolbe L. (2018). Inhibition of human tyrosinase requires molecular motifs distinctively different from mushroom tyrosinase. J. Investig. Dermatol..

[27] Halaban R., Patton R.S., Cheng E., Svedine S., Trombetta E.S., Wahl M.L., Ariyan S., Hebert D.N. (2002). Abnormal acidification of melanoma cells induces tyrosinase retention in the early secretory pathway. J. Biol. Chem..

[28] Miao F., Su M.Y., Jiang S., Luo L.F., Shi Y., Lei T.C. (2019). Intramelanocytic acidification plays a role in the antimelanogenic and antioxidative properties of vitamin c and its derivatives. Oxid. Med. Cell. Longev..

[29] Liao T., Zhou L., Liu J., Zou L., Dai T., Liu W. (2021). Inhibitory mechanism of salicylic acid on polyphenol oxidase: A cooperation between acidification and binding effects. Food Chem..

[30] Klomsakul P., Aiumsubtub A., Chalopagorn P. (2022). Evaluation of antioxidant activities and tyrosinase inhibitory effects of *Ginkgo biloba* tea extract. Sci. World J..

[31] Smeriglio A., D’Angelo V., Denaro M., Trombetta D., Raimondo F.M., Germanò M.P. (2019). Polyphenol characterization, antioxidant and skin whitening properties of *Alnus cordata* stem bark. Chem. Biodivers..

[32] Smeriglio A., D’Angelo V., Denaro M., Trombetta D., Germanò M.P. (2021). The hull of ripe pistachio nuts (*Pistacia vera* L.) as a source of new promising melanogenesis inhibitors. Plant Foods Hum. Nutr..

[33] Usuki A., Ohashi A., Sato H., Ochiai Y., Ichihashi M., Funasaka Y. (2003). The inhibitory effect of glycolic acid and lactic acid on melanin synthesis in melanoma cells. Exp. Dermatol..

[34] Knaust J., Weikard R., Albrecht E., Brunner R.M., Günther J., Kühn C. (2020). Indication of premelanosome protein (pmel) expression outside of pigmented bovine skin suggests functions beyond eumelanogenesis. Genes.

[35] Bissig C., Rochin L., van Niel G. (2016). Pmel amyloid fibril formation: The bright steps of pigmentation. Int. J. Mol. Sci..

[36] Liu Y., Chi W., Tao L., Wang G., Deepak R., Sheng L., Chen T., Feng Y., Cao X., Cheng L. (2022). Ablation of proton/glucose exporter SLC45A2 enhances melanosomal glycolysis to inhibit melanin biosynthesis and promote melanoma metastasis. J. Investig. Dermatol..

[37] Ghosh Roy S., Bakhrat A., Abdu M., Afonso S., Pereira P., Carneiro M., Abdu U. (2024). Mutations in SLC45A2 lead to loss of melanin in parrot feathers. G3.

[38] Li R., Wang Y., Liu Y., Li D., Tian Y., Liu X., Kang X., Li Z. (2023). Effects of SLC45A2 and GPNMB on melanin deposition based on transcriptome sequencing in chicken feather follicles. Animals.

[39] Galván I., Inácio Â., Nielsen Ó.K. (2017). Gyrfalcons Falco rusticolus adjust CTNS expression to food abundance: A possible contribution to cysteine homeostasis. Oecologia.

[40] Chiaverini C., Sillard L., Flori E., Ito S., Briganti S., Wakamatsu K., Fontas E., Berard E., Cailliez M., Cochat P. (2012). Cystinosin is a melanosomal protein that regulates melanin synthesis. FASEB J..

[41] Simeonov D.R., Wang X., Wang C., Sergeev Y., Dolinska M., Bower M., Fischer R., Winer D., Dubrovsky G., Balog J.Z. (2013). DNA variations in oculocutaneous albinism: An updated mutation list and current outstanding issues in molecular diagnostics. Hum. Mutat..

[42] Clark B., Elkin J., Marconi A., Turner G.F., Smith A.M., Joyce D., Miska E.A., Juntti S.A., Santos M.E. (2022). Oca2 targeting using CRISPR/Cas9 in the Malawi cichlid *Astatotilapia calliptera*. R. Soc. Open Sci..

[43] Rochin L., Hurbain I., Serneels L., Fort C., Watt B., Leblanc P., Marks M.S., De Strooper B., Raposo G., van Niel G. (2013). BACE2 processes PMEL to form the melanosome amyloid matrix in pigment cells. Proc. Natl. Acad. Sci. USA.

[44] Shimshek D.R., Jacobson L.H., Kolly C., Zamurovic N., Balavenkatraman K.K., Morawiec L., Kreutzer R., Schelle J., Jucker M., Bertschi B. (2016). Pharmacological BACE1 and BACE2 inhibition induces hair depigmentation by inhibiting PMEL17 processing in mice. Sci. Rep..

[45] Lewis R.J.S.E. (2004). Sax’s Dangerous Properties of Industrial Materials.

[46] Fuime Z. (2001). Final report on the safety assessment of malic acid and sodium malate. Int. J. Toxicol..

[47] Banerjee P., Kemmler E., Dunkel M., Preissner R. (2024). ProTox 3.0: A webserver for the prediction of toxicity of chemicals. Nucleic Acids Res..

[48] Luchian I., Goriuc A., Sandu D., Covasa M. (2022). The role of matrix metalloproteinases (MMP-8, MMP-9, MMP-13) in periodontal and peri-implant pathological processes. Int. J. Mol. Sci..

[49] Vallée A., Lecarpentier Y. (2018). Crosstalk between peroxisome proliferator-activated receptor gamma and the canonical WNT/β-catenin pathway in chronic inflammation and oxidative stress during carcinogenesis. Front. Immunol..

[50] Ashburner M., Ball C.A., Blake J.A., Botstein D., Butler H., Cherry J.M., Davis A.P., Dolinski K., Dwight S.S., Eppig J.T. (2000). Gene ontology: Tool for the unification of biology. The Gene Ontology Consortium. Nat. Genet..

[51] Kanehisa M., Sato Y., Kawashima M., Furumichi M., Tanabe M. (2016). KEGG as a reference resource for gene and protein annotation. Nucleic Acids Res..

[52] Perez-Riverol Y., Bandla C., Kundu D.J., Kamatchinathan S., Bai J., Hewapathirana S., John N.S., Prakash A., Walzer M., Wang S. (2025). The PRIDE database at 20 years: 2025 update. Nucleic Acids Res..

[53] Daina A., Michielin O., Zoete V. (2019). SwissTargetPrediction: Updated data and new features for efficient prediction of protein targets of small molecules. Nucleic Acids Res..

[54] Yao Z.J., Dong J., Che Y.J., Zhu M.F., Wen M., Wang N.N., Wang S., Lu A.P., Cao D.S. (2016). TargetNet: A web service for predicting potential drug-target interaction profiling via multi-target SAR models. J. Comput. Aided Mol. Des..

[55] Keiser M.J., Roth B.L., Armbruster B.N., Ernsberger P., Irwin J.J., Shoichet B.K. (2007). Relating protein pharmacology by ligand chemistry. Nat. Biotechnol..

[56] Stelzer G., Rosen N., Plaschkes I., Zimmerman S., Twik M., Fishilevich S., Stein T.I., Nudel R., Lieder I., Mazor Y. (2016). The GeneCards suite: From gene data mining to disease genome sequence analyses. Curr. Protoc. Bioinform..

[57] Amberger J.S., Hamosh A. (2017). Searching online mendelian inheritance in man (omim): A knowledgebase of human genes and genetic phenotypes. Curr. Protoc. Bioinform..

[58] Zhou Y., Zhang Y., Zhao D., Yu X., Shen X., Zhou Y., Wang S., Qiu Y., Chen Y., Zhu F. (2024). TTD: Therapeutic Target Database describing target druggability information. Nucleic Acids Res..

[59] Shannon P., Markiel A., Ozier O., Baliga N.S., Wang J.T., Ramage D., Amin N., Schwikowski B., Ideker T. (2003). Cytoscape: A software environment for integrated models of biomolecular interaction networks. Genome Res..

[60] Liu Y., Yang X., Gan J., Chen S., Xiao Z.X., Cao Y. (2022). CB-Dock2: Improved protein-ligand blind docking by integrating cavity detection, docking and homologous template fitting. Nucleic Acids Res..

