# Supplementary material for: Inhibition of Tyrosinase and Melanogenesis by Carboxylic Acids: Mechanistic Insights and Safety Evaluation
Source: Molecules. 2025 Apr 7;30(7):1642. doi: 10.3390/molecules30071642 (PMC11990924; doi:10.3390/molecules30071642)
Supplement: Supplementary file 1 [file molecules-30-01642-s001.zip › molecules-3563717-supplementary.pdf]

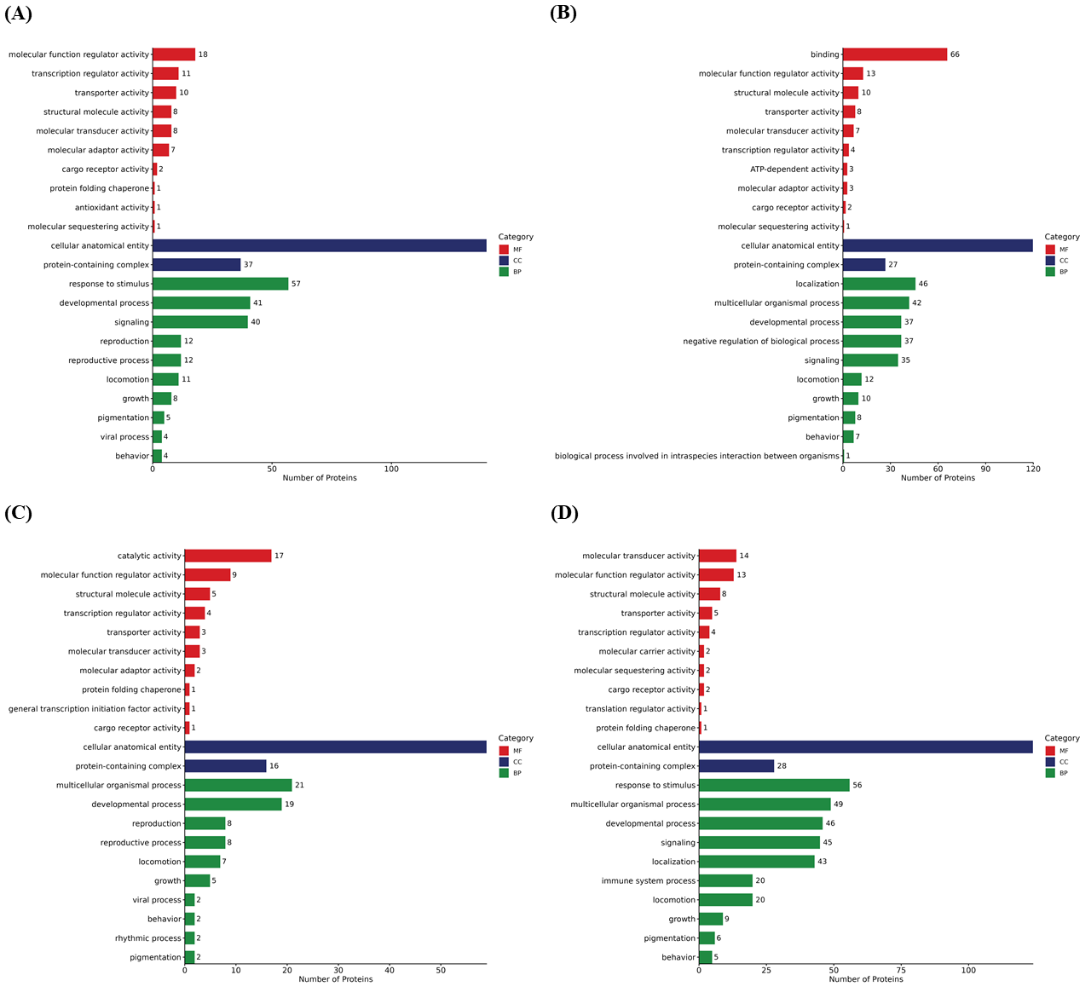

Figure S1. GO analysis was performed on differentially expressed proteins in B16-F10 cells treated with (A) 3-phenyllactic acid (6 mM), (B) lactic acid (12 mM), (C) L-pyroglutamic acid (6 mM), and (D) malic acid (6 mM).

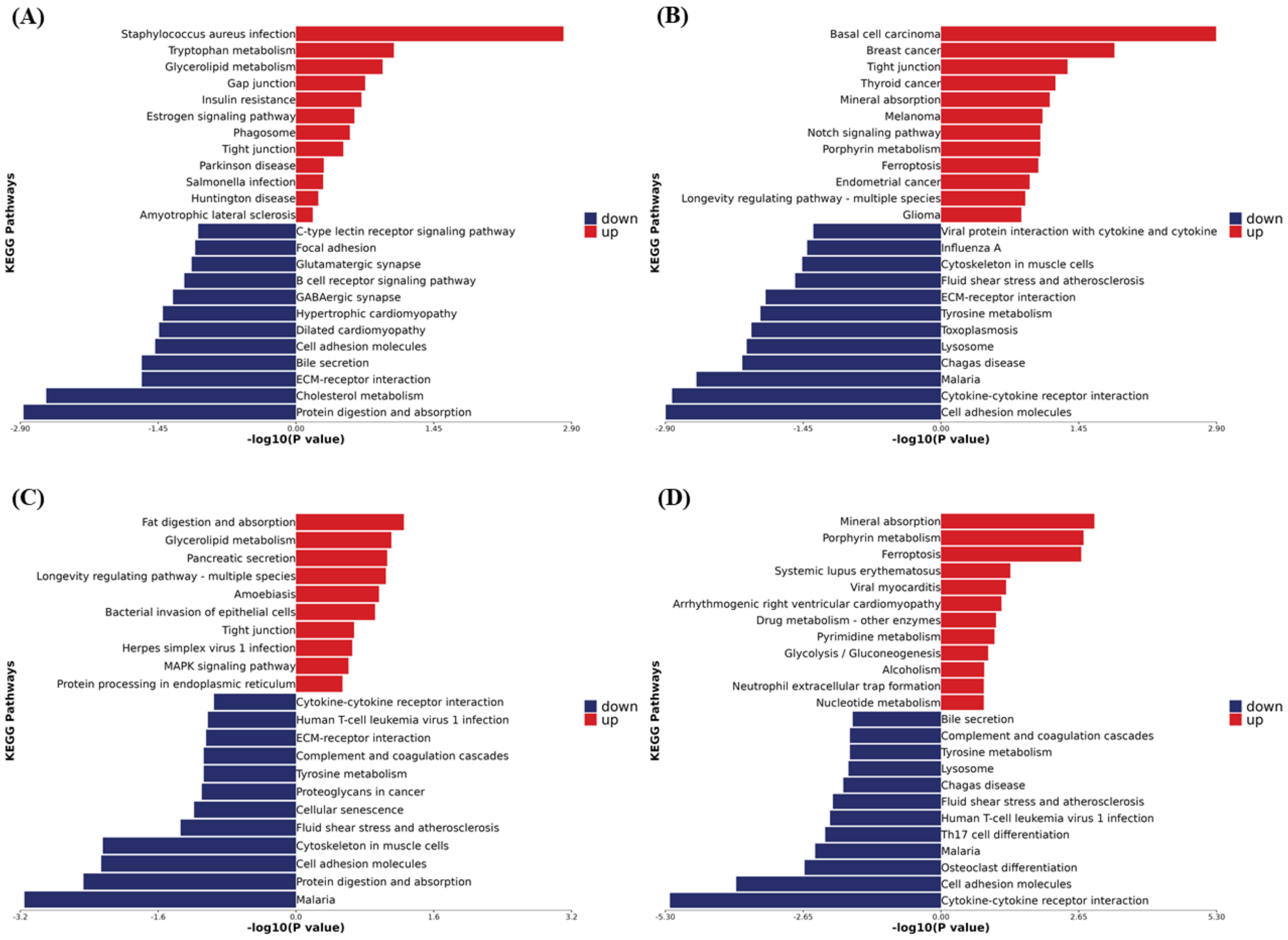

Figure S2. KEGG analysis was performed on differentially expressed proteins in B16-F10 cells treated with (A) 3-phenyllactic acid (6 mM), (B) lactic acid (12 mM), (C) L-pyroglutamic acid (6 mM), and (D) malic acid (6 mM).

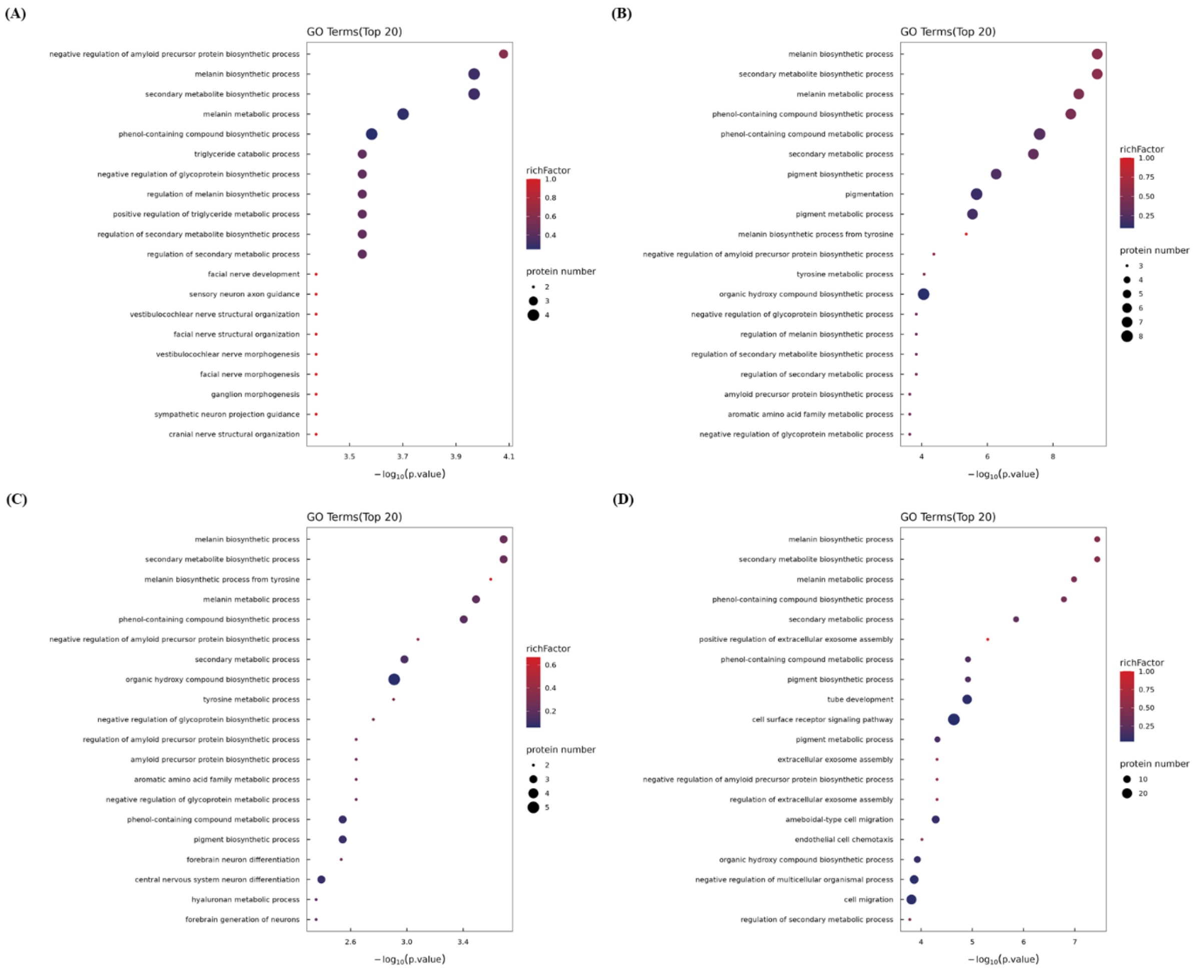

Figure S3. GO enrichment analysis (Biological Process with top 20) was performed on differentially expressed proteins in B16-F10 cells treated with (A) 3-phenyllactic acid (6 mM), (B) lactic acid (12 mM), (C) L-pyroglutamic acid (6 mM), and (D) malic acid (6 mM).

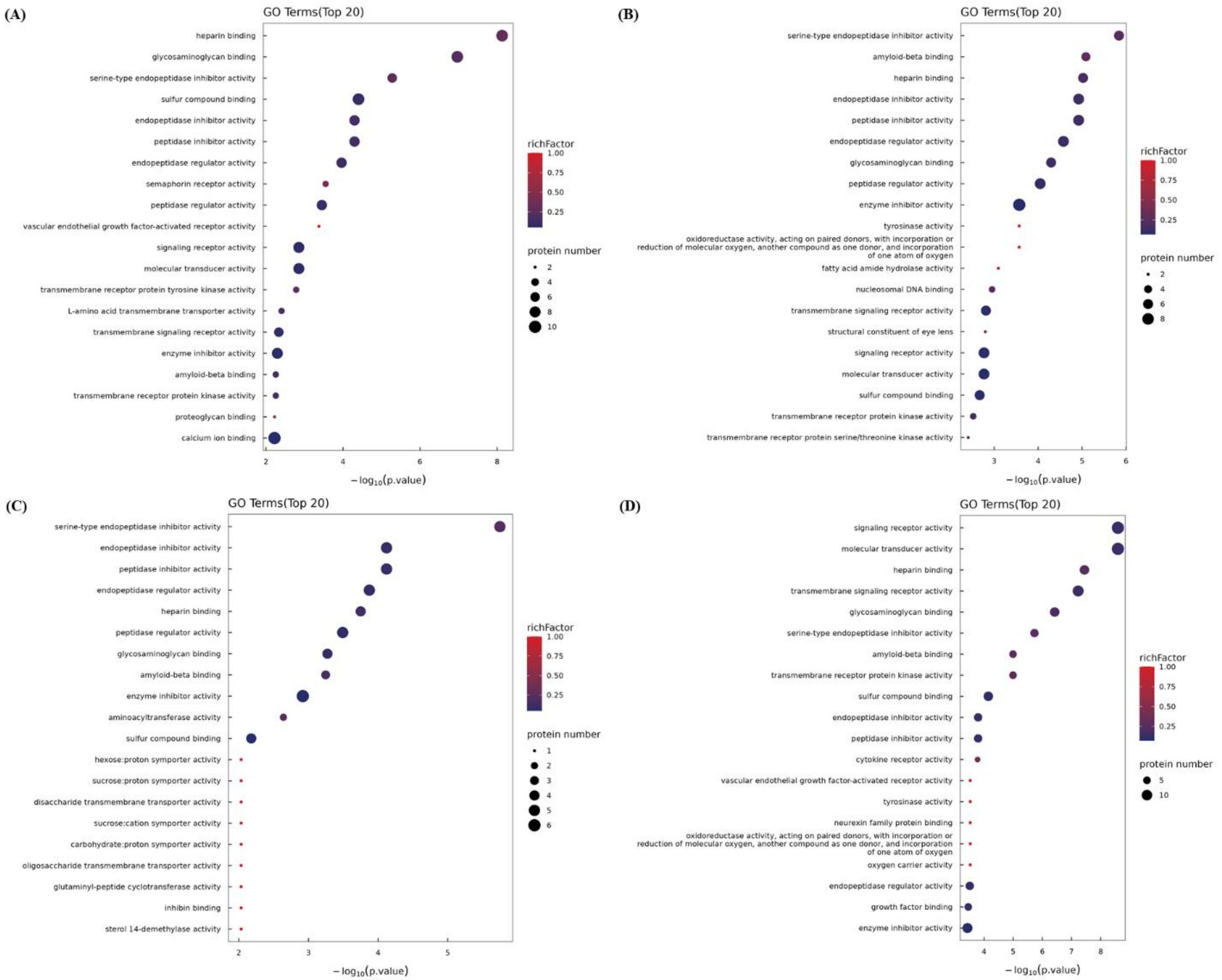

Figure S4. GO enrichment analysis (Molecular Function with top 20) was performed on differentially expressed proteins in B16-F10 cells treated with (A) 3-phenyllactic acid (6 mM), (B) lactic acid (12 mM), (C) L-pyroglutamic acid (6 mM), and (D) malic acid (6 mM).

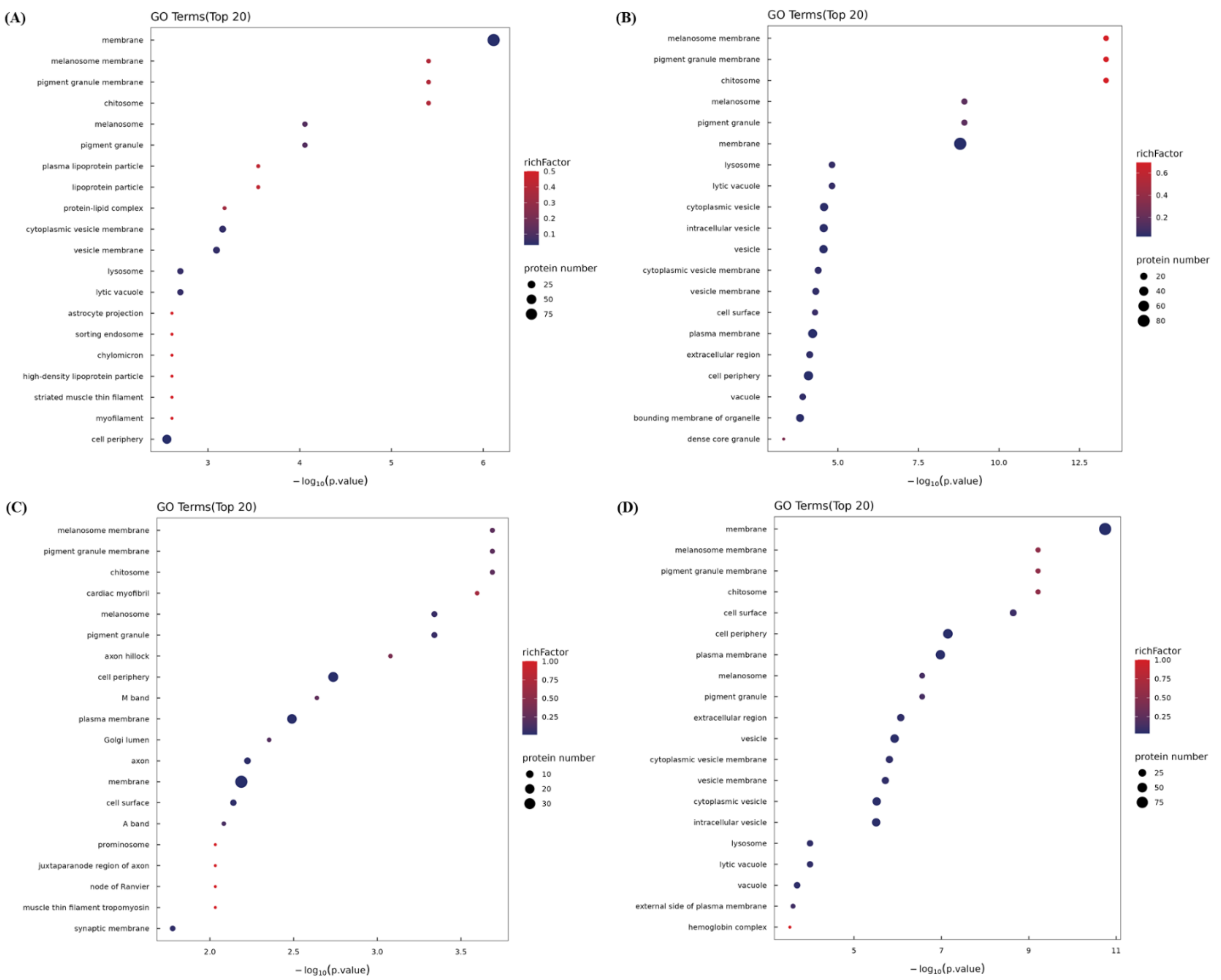

Figure S5. GO enrichment analysis (Cellular Component with top 20) was performed on differentially expressed proteins in B16-F10 cells treated with (A) 3-phenyllactic acid (6 mM), (B) lactic acid (12 mM), (C) L-pyroglutamic acid (6 mM), and (D) malic acid (6 mM).

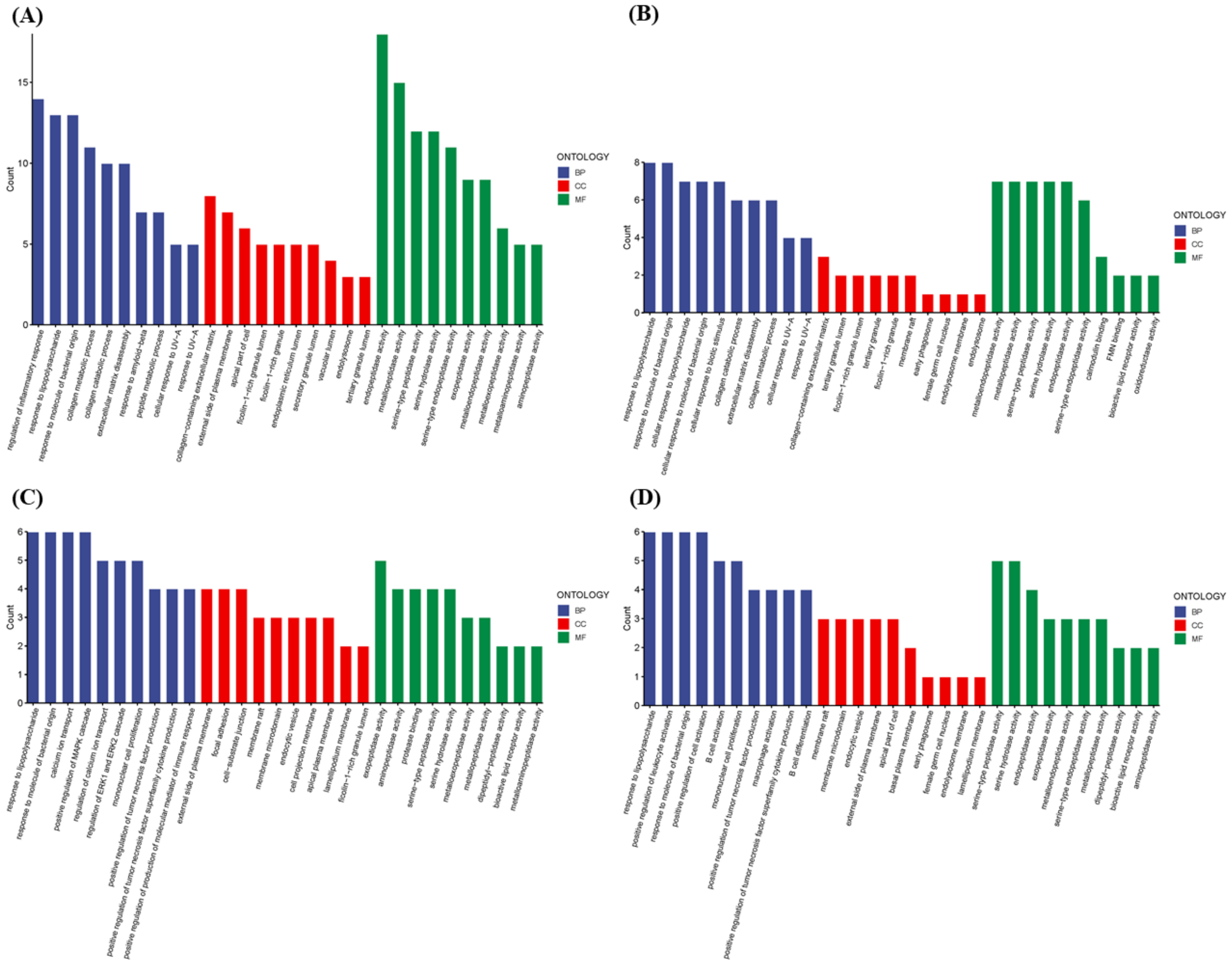

Figure S6. GO enrichment analysis was conducted to reveal the core targets associated with dermatitis-related genes and (A) 3-phenyllactic acid, (B) lactic acid, (C) L-pyroglutamic acid, and (D) malic acid.

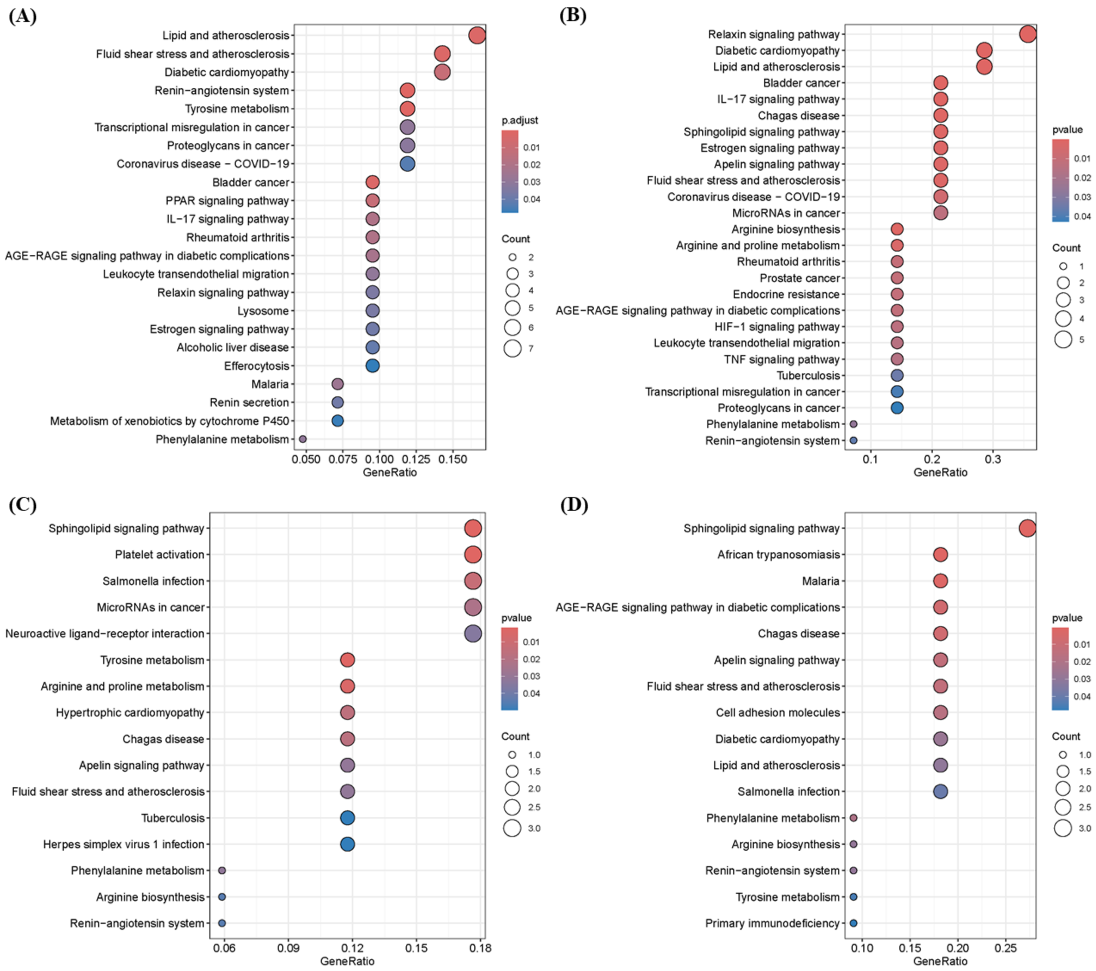

Figure S7. Enrichment analysis of KEGG pathway was conducted to reveal the core targets associated with dermatitis-related genes and (A) 3-phenyllactic acid, (B) lactic acid, (C) L-pyroglutamic acid, and (D) malic acid.

Table S1. Differentially expressed proteins, including both upregulated and downregulated proteins, were identified in B16-F10 cells by comparing the 3-phenyllactic acid treatment group to the control group.

Protein	Protein Description	Gene Name	3-Phenyllactic acid (Relative abundance)	Control (Relative abundance)	Fold change	p value
P47930	Fos-related antigen 2	Fosl2	100.571	18.3251	5.48818	0.03638
Q8C297	Transmembrane protein 14C	Tmem14c	21.7863	5.02926	4.33192	0.02975
A0A494B9Z0	40S ribosomal protein S30	Fau	2711.29	644.068	4.20963	3.2E-05
Q9D6F9	Tubulin beta-4A chain	Tubb4a	492.532	134.755	3.65501	0.04656
A6YM30	p53 (Fragment)		41.4201	12.857	3.22159	0.04123
A0A1W2P768	H3 clustered histone 14	H3c14	208.614	66.5645	3.13402	0.00951
P68134	Actin, alpha skeletal muscle	Acta1	259.144	88.703	2.92148	0.02296
A0A1Z3MI21	Ifi205	Ifi205	206.789	80.4432	2.57062	0.01828
Q8K4K2	Tribbles homolog 3	Trib3	90.7218	35.8434	2.53106	0.03899
P70318	Nucleolysin TIAR	Tial1	16.5365	6.63695	2.49158	0.01169
Q7TMH7	Sfpq protein (Fragment)	Sfpq	127.774	51.4906	2.48151	0.02156
E9Q011	E2F transcription factor 5	E2f5	57.3092	23.1772	2.47265	0.01993
Q9D219	B-cell CLL/lymphoma 9 protein	Bcl9	25.9872	11.1255	2.33583	0.0414
A0A0R4J137	Phytanoyl-CoA dioxygenase domain containing 1	Phyhd1	125.371	54.8463	2.28585	0.00147
Q91X45	Zinc finger and BTB domain-containing protein 3	Zbtb3	61.2051	26.9688	2.26948	0.02143
O88816	Serotonin N-acetyltransferase	Aanat	528.063	239.189	2.20772	0.015
Q3TMR3	FERM domain-containing protein (Fragment)	Epb41l4b	35.8917	16.4551	2.18119	0.03182

A0A0A6YXG1	non-specific serine/threonine protein kinase	Mast4	69.8537	32.9536	2.11976	0.00588
D3YX14	DNA damage-inducible transcript 3 protein (Fragment)	Ddit3	120.522	57.2556	2.10498	0.00372
Q3TCI7	L-lactate dehydrogenase	Ldha	409.972	194.843	2.10412	0.01579
A0A1D5RMC4	Bridge-like lipid transfer protein family member 1 (Fragment)	Bltp1	33.6678	16.0423	2.09868	0.0291
P10922	Histone H1.0	H1-0	507.506	246.67	2.05743	0.0027
Q3U213	Protein SERAC1	Serac1	29.0059	14.1388	2.05151	0.02208
E9PUZ3	U2 small nuclear RNA auxiliary factor 1-like 4	U2af1l4	71.1148	35.1913	2.0208	0.00638
A4FUV6	Hepatocyte growth factor receptor (Fragment)	Met	368.242	738.528	0.49862	0.00027
Q6PDX6	E3 ubiquitin-protein ligase Rnf220	Rnf220	59.6006	119.949	0.49688	0.0315
E9Q414	Apolipoprotein B-100	Apob	119.476	240.777	0.49621	0.01029
A0A1W2P6G5	Myosin, light polypeptide 6, alkali, smooth muscle and non-muscle	Myl6	14.6388	29.5658	0.49513	0.01328
P70663	SPARC-like protein 1	Sparcl1	22.8275	46.3381	0.49263	0.00367
Q3TD85	CUB domain-containing protein		55.1223	111.939	0.49243	0.00549
Q60696	Melanocyte protein PMEL	Pmel	10091.9	20529.5	0.49158	0.00054
Q9WVC3	Caveolin-2	Cav2	85.8257	174.832	0.4909	0.00242
B0LAE4	ADP-ribosylation factor-like 6 interacting protein 1 (Fragment)	Arl6ip1	183.238	373.843	0.49015	0.0307
B0V2N1	Receptor-type tyrosine-protein phosphatase S	Ptprs	373.711	763.246	0.48963	8.1E-05
A0A345IT93	Envelope polyprotein (Fragment)	env	930.455	1904.27	0.48862	0.00232

O08992	Syntenin-1	Sdcbp	1150.06	2355.5	0.48825	0.00146
P97333	Neuropilin-1	Nrp1	41.5721	85.4541	0.48648	0.01252
Q01339	Beta-2-glycoprotein 1	Apoh	85.1804	175.388	0.48567	0.02038
B2RRM5	Transmembrane emp24 domain containing 1	Tmed1	83.8584	174.674	0.48009	0.04141
A0A0R4J101	Anion exchange protein	Slc4a2	44.6504	93.0161	0.48003	0.00053
Q4FJY3	Tgoln1 protein	Tgoln1	278.3	581.043	0.47897	0.00079
A0A0G2JE26	Anthrax toxin receptor	Antxr2	38.5431	81.1982	0.47468	0.01744
Q8K209	Adhesion G-protein coupled receptor G1	Adgrg1	180.085	380.867	0.47283	3.2E-05
Q09143	High affinity cationic amino acid transporter 1	Slc7a1	108.296	229.847	0.47117	0.0083
Q3UFE8	Prosaposin	Psap	2236.98	4756.07	0.47034	0.00025
Q99J93	Interferon-induced transmembrane protein 2	Ifitm2	123.175	262.267	0.46965	0.00138
Q9WVK0	Type-1 angiotensin II receptor-associated protein	Agtrap	98.5468	210.271	0.46866	0.01538
P56390	Cyclin-dependent kinases regulatory subunit 2	Cks2	147.563	317.772	0.46437	0.02286
A0A023J653	ATP synthase protein 8	ATP8	230.465	499.097	0.46176	0.03947
P58355	Membrane-associated transporter protein	Slc45a2	1090.65	2418.41	0.45098	0.00114
H9H9R4	Glycosylated lysosomal membrane protein	Glmp	247.819	551.36	0.44947	0.01165
F6R6P2	Sarcoglycan, epsilon	Sgce	126.907	288.951	0.4392	1.9E-05
Q8VED8	Mitochondrial fission regulator 2	Mtfr2	10.3627	23.6903	0.43742	0.04052
Q9CQB5	CDGSH iron-sulfur domain-containing protein 2	Cisd2	73.2945	167.758	0.43691	0.00943
A0A0U1RNY8	Family with sequence similarity 174, member B (Fragment)	Fam174b	79.6183	183.481	0.43393	0.0078

Q3UDF0	Solute carrier family 2, facilitated glucose transporter member 6	Slc2a6	39.8104	92.4607	0.43057	0.00241
A0A0R4J1G4	RAD51 associated protein 1	Rad51ap1	31.2223	72.7643	0.42909	0.00264
A2ATP8	Solute carrier family 24, member 5	Slc24a5	53.6905	125.278	0.42857	0.01017
P11370	Retrovirus-related Env polyprotein from Fv-4 locus	Fv4	141.515	338.768	0.41773	0.0032
G3X9X1	Kelch repeat and BTB domain-containing protein 2	Kbtbd2	28.6675	68.6353	0.41768	0.02024
A0A0R4J1M6	DiGeorge syndrome critical region gene 2	Dgcr2	62.4025	150.48	0.41469	0.00176
H6WCS3	Interleukin-6 signal transductor	il6st	39.4679	95.8577	0.41173	0.00852
A0A0R4J1C7	Microfibrillar-associated protein 3	Mfap3	61.8879	151.399	0.40877	0.00778
A9CLV6	Axotomy induced glycoprotein 2	Serinc1	481.151	1177.56	0.4086	0.00033
Q8BM72	Heat shock 70 kDa protein 13	Hspa13	214.369	524.896	0.4084	0.00204
A0A0R4J0I8	Beta-site APP-cleaving enzyme 2	Bace2	80.1446	196.978	0.40687	0.00147
P35951	Low-density lipoprotein receptor	Ldlr	318.342	792.332	0.40178	4E-06
Q3TCT4	Ectonucleoside triphosphate diphosphohydrolase 7	Entpd7	19.8938	49.7636	0.39976	0.00899
Q8R2Q8	Bone marrow stromal antigen 2	Bst2	21.4294	54.2614	0.39493	0.04386
Q3UJQ2	Hydroxymethylglutaryl-coenzyme A synthase C-terminal domain-containing protein (Fragment)	Hmgcs1	29.4265	75.1459	0.39159	0.02618
A8DUN2	Beta-globin	Hbbt1	563.255	1451.88	0.38795	0.02288
P32261	Antithrombin-III	Serpinc1	64.5042	170.753	0.37776	0.01158

Q3TU35	Fibronectin type-III domain-containing protein	Il10rb	89.0782	236.742	0.37627	0.00025
A0A654IDR4	Gap junction protein	Cxne	54.1017	144.165	0.37528	0.00079
Q3UN36	Glycoprotein integral membrane 1	Ginm1	57.0973	152.623	0.37411	0.0187
Q8CFE6	Sodium-coupled neutral amino acid symporter 2	Slc38a2	432.069	1157.31	0.37334	0.00013
A0A2I3BPT1	Amyloid-beta A4 protein	App	358.387	961.22	0.37285	0.00025
Q8BHL7	CDC42 small effector protein 1	Cdc42se1	70.5782	191.615	0.36833	0.0246
A0A2I3BQL3	Lysosomal-associated protein transmembrane 4B (Fragment)	Laptm4b	117.849	321.192	0.36691	0.01131
F7A1B4	Endoglin	Eng	269.266	740.794	0.36348	0.00375
P86046	Inward rectifier potassium channel 13	Kcnj13	97.8063	269.546	0.36286	0.03372
D3Z5K6	DnaJ heat shock protein family (Hsp40) member C19	Dnajc19	84.9712	236.914	0.35866	0.00622
P07147	5,6-dihydroxyindole-2-carboxylic acid oxidase	Tyrp1	6554.48	18320.4	0.35777	0.00038
A0A2I3BRQ3	Inter-alpha trypsin inhibitor, heavy chain 3	Itih3	54.9768	155.799	0.35287	0.00158
F6SPK0	Ubiquitin-conjugating enzyme E2J 1 (Fragment)	Ube2j1	12.6354	35.9532	0.35144	0.00438
Q3TJQ2	Adenosine 3'-phospho 5'-phosphosulfate transporter 1	Slc35b2	85.8437	246.98	0.34757	0.04505
Q8K0C4	Lanosterol 14-alpha demethylase	Cyp51a1	679.607	1970.18	0.34495	0.00233
Q91VK4	Integral membrane protein 2C	Itm2c	168.932	494.545	0.34159	4E-06
O35375	Neuropilin-2	Nrp2	303.693	891.148	0.34079	6.4E-05
A0A2R8W6S4	Protein kinase C and casein kinase substrate in neurons 2 (Fragment)	Pacsin2	10.2342	30.0762	0.34028	0.04963

A0A494BAA4	Nedd4 family interacting protein 1	Ndfip1	143.654	436.11	0.3294	0.00016
Q3UGA5	Tetraspanin 10	Tspan10	60.3455	183.874	0.32819	3.3E-05
A0A1L1SRJ4	Tetraspanin 3	Tspan3	71.034	224.64	0.31621	0.00057
P40749	Synaptotagmin-4	Syt4	82.4893	265.614	0.31056	3.5E-05
P57757	Cystinosin	Ctns	52.0249	168.541	0.30868	0.01937
Q792Z1	Trypsin 10	Try10	515.681	1774.46	0.29061	0.02186
Q3UYL1	Ubiquitin-like protein	Ubl3	347.807	1233.98	0.28186	0.00393
Q9WVA4	Transgelin-2	Tagln2	27.0292	96.0225	0.28149	0.0098
Q2TA50	Melan-A	Mlana	478.302	1894.7	0.25244	3.2E-05
P18828	Syndecan-1	Sdc1	38.5414	157.925	0.24405	0.01161
O89051	Integral membrane protein 2B	Itm2b	102.885	423.816	0.24276	3.7E-06
B5B2P6	Nuclear factor of activated T-cells c2 isoform IA-IIL-Xa	Nfatc2	10.7114	45.2806	0.23656	0.02215
D3YVM2	Transmembrane protein 59	Tmem59	335.887	1441.39	0.23303	0.00033
A0A0N4SVU8	Transmembrane protein 176A (Fragment)	Tmem176a	56.1503	246.627	0.22767	0.00073
Q3UDL6	Amyloid-like protein 2	Aplp2	86.1003	383.288	0.22464	1.1E-05
Q60961	Lysosomal-associated transmembrane protein 4A	Laptm4a	72.9659	351.45	0.20761	0.00071
Q9R1Q6	Transmembrane protein 176B	Tmem176b	151.647	886.929	0.17098	8.2E-06

Table S2. Differentially expressed proteins, including both upregulated and downregulated proteins, were identified in B16-F10 cells by comparing the lactic acid treatment group to the control group.

Protein	Protein Description	Gene Name	Lactic acid	Control	Fold change	p value
			(Relative abundance)			
Q14A47	Coiled-coil domain containing 72	Tma7	91.0211	20.6339	4.41124	0.02315
A0A1Y7VIN9	Zinc finger, AN1-type domain 4	Zfand4	772.634	214.592	3.60048	0.00669
A0A494B9Z0	40S ribosomal protein S30	Fau	2084.7	644.068	3.23678	0.01801
P48316	Growth arrest and DNA damage-inducible protein GADD45 alpha	Gadd45a	123.735	39.3491	3.14454	0.00325
D3YX14	DNA damage-inducible transcript 3 protein (Fragment)	Ddit3	174.722	57.2556	3.05162	0.00015
P23927	Alpha-crystallin B chain	Cryab	973.168	344.043	2.82862	2.2E-05
B1AQJ3	Tissue inhibitor of metalloproteinase 2 (Fragment)	Timp2	13.4381	4.87483	2.75664	0.00545
Q3TMR3	FERM domain-containing protein (Fragment)	Epb41l4b	44.609	16.4551	2.71095	0.0049
D6RHC1	Autophagy related 16-like 2 (S. cerevisiae)	Atg16l2	20.9545	7.80598	2.68442	0.00588
P43275	Histone H1.1	H1-1	116.235	46.617	2.49341	0.01425
O08967	Cytohesin-3	Cyth3	24.0969	9.72601	2.47758	0.01852
Q8BG95	Protein phosphatase 1 regulatory subunit 12B	Ppp1r12b	41.079	17.0671	2.40691	0.00783
Q4FJW1	Activating transcription factor 3	Atf3	260.308	108.938	2.38951	0.04212
P51141	Segment polarity protein dishevelled homolog DVL-1	Dvl1	29.2826	12.267	2.3871	0.01483
Q9JK83	Partitioning defective 6 homolog beta	Pard6b	62.6642	26.7555	2.3421	0.01198

Q14DK5	HHIP-like protein 1	Hhipl1	71.3025	30.7978	2.31518	0.00268
Q3UT72	Oxidative stress-responsive serine-rich protein 1	Oser1	51.9866	23.4578	2.21617	0.0018
P15864	Histone H1.2	H1-2	8421.78	3956.43	2.12863	0.00161
Q8K387	Ubiquitin carboxyl-terminal hydrolase 45	Usp45	131.614	61.8606	2.1276	0.03846
P10922	Histone H1.0	H1-0	513.974	246.67	2.08365	0.00242
Q8K4K2	Tribbles homolog 3	Trib3	74.3084	35.8434	2.07314	0.01116
P84104	Serine/arginine-rich splicing factor 3	Srsf3	2587.13	1259.31	2.0544	0.03361
P09528	Ferritin heavy chain	Fth1	2181.32	1062.4	2.0532	0.00193
Q3TCT4	Ectonucleoside triphosphate diphosphohydrolase 7	Entpd7	24.6614	49.7636	0.49557	0.02336
Q62179	Semaphorin-4B	Sema4b	54.6387	110.551	0.49424	0.00107
Q0VGR4	Multiple EGF-like-domains 9	Megf9	11.0552	22.4145	0.49322	0.00364
Q4FJL1	receptor protein serine/threonine kinase	Tgfbr1	100.947	206.07	0.48987	0.00778
Q60696	Melanocyte protein PMEL	Pmel	10052.9	20529.5	0.48968	0.00054
Q3TP92	CTD nuclear envelope phosphatase 1	Ctdnep1	110.502	227.867	0.48494	0.00134
Q3TD85	CUB domain-containing protein		54.0726	111.939	0.48306	0.00204
Q3TZF1	receptor protein serine/threonine kinase	Acvr1b	21.9185	45.466	0.48209	0.01379
Q3TAV1	PKD domain-containing protein	Gpnmb	3950.72	8206.46	0.48142	0.00017
Q69ZP3	Probable hydrolase PNKD	Pnkd	76.2304	159.441	0.47811	0.0073
A0A0R4J1M6	DiGeorge syndrome critical region gene 2	Dgcr2	71.6577	150.48	0.4762	0.00131
A2RTI3	Legumain	Lgmn	256.992	559.171	0.45959	0.00075
F8WIU9	Small integral membrane protein 7	Smim7	50.7959	110.692	0.45889	3.8E-05
Q7TPQ9	Arrestin domain-containing protein 3	Arrdc3	31.8403	69.8237	0.45601	0.00623

P58355	Membrane-associated transporter protein	Slc45a2	1101.74	2418.41	0.45557	0.00306
P25118	Tumor necrosis factor receptor superfamily member 1A	Tnfrsf1a	59.2152	131.287	0.45104	0.0019
O89023	Tripeptidyl-peptidase 1	Tpp1	657.354	1471.57	0.4467	0.00458
A2ATP8	Solute carrier family 24, member 5	Slc24a5	55.9377	125.278	0.44651	0.01476
A0A2I3BRQ3	Inter-alpha trypsin inhibitor, heavy chain 3	Itih3	68.8744	155.799	0.44207	0.00769
Q62052	P protein	Oca2	85.702	194.361	0.44094	0.00249
Q6ZPK5	MKIAA1646 protein (Fragment)	Cerk	35.2856	83.1742	0.42424	0.01691
B0V2N1	Receptor-type tyrosine-protein phosphatase S	Ptprs	320.817	763.246	0.42033	5.6E-05
A0A1L1SRJ4	Tetraspanin 3	Tspan3	94.0887	224.64	0.41884	0.00405
Q8K0B2	Lysosomal cobalamin transport escort protein LMBD1	Lmbrd1	250.798	607.345	0.41294	0.00324
B2CY76	Dopachrome tautomerase	Dct	8017.08	19593.9	0.40916	0.00015
P11344	Tyrosinase	Tyr	4950.94	12192.7	0.40606	7.5E-05
P32261	Antithrombin-III	Serpinc1	66.0863	170.753	0.38703	0.01571
Q3UN36	Glycoprotein integral membrane 1	Ginm1	58.5354	152.623	0.38353	0.01843
A0A0R4J0I8	Beta-site APP-cleaving enzyme 2	Bace2	75.4985	196.978	0.38328	0.002
P35951	Low-density lipoprotein receptor	Ldlr	300.171	792.332	0.37885	4.6E-05
H3BJ77	FUN14 domain containing 1	Fundc1	24.6651	65.8104	0.37479	0.02691
P40749	Synaptotagmin-4	Syt4	98.87	265.614	0.37223	0.00016
A0A2I3BPT1	Amyloid-beta A4 protein	App	357.374	961.22	0.37179	0.00027
O35375	Neuropilin-2	Nrp2	329.555	891.148	0.36981	7.4E-05
A0A7G9TMG4	Gag	gag	82.7643	227.977	0.36304	0.00288

A9CLV6	Axotomy induced glycoprotein 2	Serinc1	424.756	1177.56	0.36071	5.9E-05
Q91XX7	Protocadherin gamma B2	Pcdhgb2	249.627	695.605	0.35886	0.00466
A0A0U1RNY8	Family with sequence similarity 174, member B (Fragment)	Fam174b	65.445	183.481	0.35669	0.0053
Q8K0C4	Lanosterol 14-alpha demethylase	Cyp51a1	679.296	1970.18	0.34479	0.00254
Q3TU35	Fibronectin type-III domain-containing protein	Il10rb	77.7889	236.742	0.32858	0.00014
A0A654IDR4	Gap junction protein	Cxne	45.7862	144.165	0.3176	0.00013
Q91VK4	Integral membrane protein 2C	Itm2c	151.934	494.545	0.30722	6.8E-06
Q99J93	Interferon-induced transmembrane protein 2	Ifitm2	80.3485	262.267	0.30636	0.00081
Q3UYL1	Ubiquitin-like protein	Ubl3	376.047	1233.98	0.30474	0.00562
P57757	Cystinosin	Ctns	50.6162	168.541	0.30032	0.01518
F6R6P2	Sarcoglycan, epsilon	Sgce	86.6233	288.951	0.29979	2.3E-05
A0A0N4SVU8	Transmembrane protein 176A (Fragment)	Tmem176a	73.4436	246.627	0.29779	0.00126
A0A0R4J1C7	Microfibrillar-associated protein 3	Mfap3	43.5516	151.399	0.28766	0.00336
Q14AE4	WAP four-disulfide core domain protein 3	Wfdc3	13.7729	51.1582	0.26922	0.00497
Q8CFE6	Sodium-coupled neutral amino acid symporter 2	Slc38a2	301.071	1157.31	0.26015	8.7E-05
P18828	Syndecan-1	Sdc1	41.0815	157.925	0.26013	0.01022
Q3UDL6	Amyloid-like protein 2	Aplp2	97.1543	383.288	0.25348	3.3E-05
P07147	5,6-dihydroxyindole-2-carboxylic acid oxidase	Tyrp1	4381.52	18320.4	0.23916	0.00014
Q3UGA5	Tetraspanin 10	Tspan10	41.7138	183.874	0.22686	7.6E-05
A0A494BAA4	Nedd4 family interacting protein 1	Ndfip1	96.0609	436.11	0.22027	7.2E-05
O89051	Integral membrane protein 2B	Itm2b	85.3483	423.816	0.20138	3.9E-06

Q2TA50	Melan-A	Mlana	344.364	1894.7	0.18175	4.6E-05
D3YVM2	Transmembrane protein 59	Tmem59	230.074	1441.39	0.15962	0.00026
Q9R1Q6	Transmembrane protein 176B	Tmem176b	140.497	886.929	0.15841	6E-06
A0A2I3BQL3	Lysosomal-associated protein transmembrane 4B (Fragment)	Laptm4b	49.2481	321.192	0.15333	0.0021
Q60961	Lysosomal-associated transmembrane protein 4A	Laptm4a	49.949	351.45	0.14212	0.0005

Table S3. Differentially expressed proteins, including both upregulated and downregulated proteins, were identified in B16-F10 cells by comparing the L-pyroglutamic acid treatment group to the control group.

Protein	Protein Description	Gene Name	L-Pyroglutamic acid	Control	Fold change	p value
			(Relative abundance)			
P23927	Alpha-crystallin B chain	Cryab	1027.18	344.043	2.9856	8E-06
A0A0G2JFK5	[histone H4]-lysine(20) N-methyltransferase	Kmt5a	54.0024	20.2298	2.66945	0.04598
P43275	Histone H1.1	H1-1	119.413	46.617	2.56157	0.0147
Q3TMR3	FERM domain-containing protein (Fragment)	Epb41l4b	38.1861	16.4551	2.32062	0.02216
A0A0G2LB90	Tubulin tyrosine ligase-like family, member 7	Ttll7	70.5025	32.0579	2.19922	0.00924
Q8BH73	Glutaminyl-peptide cyclotransferase-like protein	Qpctl	223.689	102.929	2.17324	0.04717
A0A494B9Z0	40S ribosomal protein S30	Fau	1394.24	644.068	2.16474	0.014
Q9QYS0	ATP sulfurylase/APS kinase isoform SK2		32.3712	15.1613	2.13511	0.04637
P14602	Heat shock protein beta-1	Hspb1	327.238	157.418	2.07878	0.00672
Q3TZF1	receptor protein serine/threonine kinase	Acvr1b	22.6124	45.466	0.49735	0.01566
O35375	Neuropilin-2	Nrp2	442.209	891.148	0.49622	0.00028
B2CY76	Dopachrome tautomerase	Dct	9611.79	19593.9	0.49055	6.6E-05
A0A654IDR4	Gap junction protein	Cxne	70.0271	144.165	0.48574	0.00052
F6VZH1	Small G protein signaling modulator 3 (Fragment)	Sgsm3	50.8052	105.579	0.4812	0.03246
Q9JIY7	N-acetyltransferase 8	Nat8	81.6093	169.813	0.48058	0.02917
Q91VK4	Integral membrane protein 2C	Itm2c	237.268	494.545	0.47977	0.00018
P58355	Membrane-associated transporter protein	Slc45a2	1154.4	2418.41	0.47734	0.00172

A0A494BAA4	Nedd4 family interacting protein 1	Ndfip1	207.063	436.11	0.4748	0.00038
F6R6P2	Sarcoglycan, epsilon	Sgce	137.163	288.951	0.47469	6.5E-05
Q8K0C8	Cytochrome c oxidase assembly protein COX19	Cox19	122.913	260.19	0.4724	0.00135
A0A2I3BRQ3	Inter-alpha trypsin inhibitor, heavy chain 3	Itih3	73.5586	155.799	0.47214	0.00201
Q3TU35	Fibronectin type-III domain-containing protein	Il10rb	110.214	236.742	0.46555	0.00031
P18828	Syndecan-1	Sdc1	70.8217	157.925	0.44845	0.022
P43407	Syndecan-2	Sdc2	32.0725	73.5237	0.43622	0.03717
Q2TA50	Melan-A	Mlana	825.807	1894.7	0.43585	0.00022
P32261	Antithrombin-III	Serpinc1	73.0968	170.753	0.42808	0.01947
P07147	5,6-dihydroxyindole-2-carboxylic acid oxidase	Tyrp1	7507.16	18320.4	0.40977	0.00082
Q8CFE6	Sodium-coupled neutral amino acid symporter 2	Slc38a2	467.189	1157.31	0.40369	0.0002
O89051	Integral membrane protein 2B	Itm2b	169.102	423.816	0.399	4.5E-05
Q8K0C4	Lanosterol 14-alpha demethylase	Cyp51a1	784.401	1970.18	0.39814	0.00336
Q3UYL1	Ubiquitin-like protein	Ubl3	475.5	1233.98	0.38534	0.00876
P86046	Inward rectifier potassium channel 13	Kcnj13	100.427	269.546	0.37258	0.0166
Q3UDL6	Amyloid-like protein 2	Aplp2	142.311	383.288	0.37129	3.4E-05
A0A5F8MPN1	FRY microtubule binding protein	Fry	448.946	1214.64	0.36961	0.02016
A0A140LI36	Androglobin	Adgb	14.6273	40.8562	0.35802	0.01935
Q3UGA5	Tetraspanin 10	Tspan10	65.7562	183.874	0.35762	7E-05
D3YVM2	Transmembrane protein 59	Tmem59	506.09	1441.39	0.35111	0.00069
Q9R1Q6	Transmembrane protein 176B	Tmem176b	303.108	886.929	0.34175	6.2E-05

B5B2P6	Nuclear factor of activated T-cells c2 isoform IA-IIL-Xa	Nfatc2	15.3719	45.2806	0.33948	0.03551
A0A2I3BQL3	Lysosomal-associated protein transmembrane 4B (Fragment)	Laptm4b	100.749	321.192	0.31367	0.02597
Q60961	Lysosomal-associated transmembrane protein 4A	Laptm4a	93.3288	351.45	0.26555	0.0029

Table S4. Differentially expressed proteins, including both upregulated and downregulated proteins, were identified in B16-F10 cells by comparing the malic acid treatment group to the control group.

Protein	Protein Description	Gene Name	Malic acid	Control	Fold change	p value
			(Relative abundance)			
B2RWH3	Histone H2A	Hist2h2aa1	235.384	42.3404	5.55932	0.0371
A0A494B9Z0	40S ribosomal protein S30	Fau	2709.28	644.068	4.20651	0.00478
Q5DTP0	MKIAA4087 protein (Fragment)	Lama2	16.2639	5.91755	2.74842	0.03986
Q9CPX4	Ferritin	Ftl1	14968.3	5937.83	2.52083	0.00066
D3Z468	PWWP domain containing 3A, DNA repair factor (Fragment)	Pwwp3a	31.631	12.5982	2.51076	0.03314
Q8BG95	Protein phosphatase 1 regulatory subunit 12B	Ppp1r12b	42.6323	17.0671	2.49792	0.01222
P43275	Histone H1.1	H1-1	111.277	46.617	2.38704	0.01073
P43024	Cytochrome c oxidase subunit 6A1, mitochondrial	Cox6a1	970.326	416.261	2.33105	0.00254
P09528	Ferritin heavy chain	Fth1	2395.92	1062.4	2.2552	0.00307
D3YZU6	Hydroxyacylglutathione hydrolase-like	Haghl	204.564	97.8168	2.0913	0.00209
P10922	Histone H1.0	H1-0	515.724	246.67	2.09074	0.0315
E9Q011	E2F transcription factor 5	E2f5	47.5601	23.1772	2.05202	0.00114
D9J2V6	Pre B cell leukemia homeobox 1	Pbx1	12.4222	6.09454	2.03824	0.02108
P04184	Thymidine kinase, cytosolic	Tk1	154.578	76.7047	2.01523	0.00537
F7A1B4	Endoglin	Eng	368.232	740.794	0.49708	0.00859
P57757	Cystinosin	Ctns	83.6286	168.541	0.49619	0.0407
O35407	receptor protein-tyrosine kinase	Ddr1	34.2622	69.1324	0.4956	0.00246

Q3UFE8	Prosaposin	Psap	2355.1	4756.07	0.49518	0.00047
E9PW22	Abhydrolase domain containing 14A	Abhd14a	14.2333	28.7735	0.49467	0.03611
Q99LJ8	Dehydrodolichyl diphosphate synthase complex subunit Nus1	Nus1	18.5805	37.6211	0.49389	0.00431
P21956	Lactadherin	Mfge8	56.9975	116.612	0.48878	0.00021
Q8BPF4	Globin family profile domain-containing protein		365.562	751.032	0.48675	0.00107
Q3TD49	Signal peptide peptidase-like 2B	Sppl2b	55.4764	114.222	0.48569	0.03314
Q3TZU6	Rho-related GTP-binding protein RhoJ	Rhoj	32.9415	68.1397	0.48344	0.00498
P97333	Neuropilin-1	Nrp1	41.1516	85.4541	0.48156	0.00939
Q3UFC9	PH domain-containing protein	Plekhb2	69.1438	143.701	0.48116	0.00227
Q8R001	Microtubule-associated protein RP/EB family member 2	Mapre2	22.0102	46.0962	0.47748	0.02074
P15261	Interferon gamma receptor 1	Ifngr1	52.7412	110.571	0.47699	0.00644
A0A0R4J1M6	DiGeorge syndrome critical region gene 2	Dgcr2	71.5753	150.48	0.47565	0.00128
O08992	Syntenin-1	Sdcbp	1110.96	2355.5	0.47164	0.00147
Q3UKC1	Tax1-binding protein 1 homolog	Tax1bp1	224.543	476.255	0.47148	3.4E-06
Q4FJL1	receptor protein serine/threonine kinase	Tgfbr1	97.0863	206.07	0.47113	0.00465
B0V2N1	Receptor-type tyrosine-protein phosphatase S	Ptprs	355.762	763.246	0.46612	5.1E-05
O70401	Tetraspanin-6	Tspan6	78.2052	167.829	0.46598	0.01123
P25118	Tumor necrosis factor receptor superfamily member 1A	Tnfrsf1a	60.8853	131.287	0.46376	0.00053
M0QWC2	Microtubule-associated protein 1 light chain 3 beta	Map1lc3b	340.987	736.945	0.4627	0.00214
D3Z3F1	Pre-mRNA 3'-end-processing factor FIP1 (Fragment)	Fip1l1	8.47551	18.3507	0.46186	0.01888
Q1XID4	Renin receptor	Atp6ap2	687.832	1490.38	0.46151	0.00018

A2AW73	Sema domain, transmembrane domain (TM), and cytoplasmic domain, (semaphorin) 6D	Sema6d	37.0731	80.4988	0.46054	0.03763
Q8K0C4	Lanosterol 14-alpha demethylase	Cyp51a1	905.416	1970.18	0.45956	0.00635
Q60696	Melanocyte protein PMEL	Pmel	9402.65	20529.5	0.45801	0.00038
B2CY76	Dopachrome tautomerase	Dct	8876.2	19593.9	0.45301	2.5E-05
Q62179	Semaphorin-4B	Sema4b	49.4916	110.551	0.44768	0.00063
Q3TJA0	E3 ubiquitin-protein ligase pellino homolog	Peli1	22.5108	50.9468	0.44185	0.0228
Q8K0B2	Lysosomal cobalamin transport escort protein LMBD1	Lmbrd1	266.144	607.345	0.43821	0.00388
P23927	Alpha-crystallin B chain	Cryab	149.741	344.043	0.43524	0.00015
Q60943	Interleukin-17 receptor A	Il17ra	50.3732	115.849	0.43482	0.00407
P62746	Rho-related GTP-binding protein RhoB	Rhob	111.022	255.768	0.43407	0.00066
A0A0U1RNY8	Family with sequence similarity 174, member B (Fragment)	Fam174b	79.5232	183.481	0.43341	0.0075
A0A571BES6	Ring finger protein 130	Rnf130	18.7039	43.2453	0.43251	0.00294
P14602	Heat shock protein beta-1	Hspb1	68.0117	157.418	0.43204	0.01534
A1A549	Transcription factor 3	Tcf7l1	120.057	280.097	0.42863	0.00239
Q3TAV1	PKD domain-containing protein	Gpnmb	3501.65	8206.46	0.42669	6.3E-05
Q00493	Carboxypeptidase E	Cpe	138.512	325.92	0.42499	0.00014
Q8VBZ0	Dehydrogenase/reductase SDR family member on chromosome X homolog	Dhrsx	38.5911	91.3664	0.42238	0.00189
P58355	Membrane-associated transporter protein	Slc45a2	1018.77	2418.41	0.42126	0.00162
Q8QZY6	Tetraspanin-14	Tspan14	33.9599	81.4113	0.41714	0.04022
A0A0R4J101	Anion exchange protein	Slc4a2	38.5146	93.0161	0.41406	0.00022

A2ATP8	Solute carrier family 24, member 5	Slc24a5	51.7762	125.278	0.41329	0.00744
P32261	Antithrombin-III	Serpinc1	69.6929	170.753	0.40815	0.01323
Q9CQW9	Interferon-induced transmembrane protein 3	Ifitm3	79.7955	197.009	0.40504	0.00078
A0A654IDR4	Gap junction protein	Cxne	58.2879	144.165	0.40431	0.00185
O35375	Neuropilin-2	Nrp2	353.134	891.148	0.39627	9.5E-05
O35566	CD151 antigen	Cd151	84.9898	223.867	0.37964	0.01069
P11344	Tyrosinase	Tyr	4585.6	12192.7	0.37609	8.9E-06
A0A0U1RPL0	Ataxin 2-like	Atxn2l	819.309	2189.16	0.37426	0.00031
E9QJU7	SH3-domain binding protein 2	Sh3bp2	29.7068	80.307	0.36991	0.00228
H6WCS3	Interleukin-6 signal transductor	il6st	34.8612	95.8577	0.36368	0.00668
A2RTI3	Legumain	Lgmn	199.791	559.171	0.3573	0.00023
A8DUN2	Beta-globin	Hbbt1	513.548	1451.88	0.35371	0.00171
Q3TZF1	receptor protein serine/threonine kinase	Acvr1b	15.8239	45.466	0.34804	0.00551
Q14AE4	WAP four-disulfide core domain protein 3	Wfdc3	17.4078	51.1582	0.34027	0.02629
P35951	Low-density lipoprotein receptor	Ldlr	260.354	792.332	0.32859	2.9E-06
Q8K209	Adhesion G-protein coupled receptor G1	Adgrg1	123.959	380.867	0.32547	7.9E-07
Q3TD85	CUB domain-containing protein		35.7051	111.939	0.31897	0.00204
A0A1L1SRJ4	Tetraspanin 3	Tspan3	69.5866	224.64	0.30977	0.00064
A0A2I3BPT1	Amyloid-beta A4 protein	App	297.057	961.22	0.30904	0.00017
O89023	Tripeptidyl-peptidase 1	Tpp1	446.269	1471.57	0.30326	0.0017
A0A0R4J0I8	Beta-site APP-cleaving enzyme 2	Bace2	58.0941	196.978	0.29493	0.00058
Q91XX7	Protocadherin gamma B2	Pcdhgb2	196.59	695.605	0.28262	0.00259

Q3TU35	Fibronectin type-III domain-containing protein	Il10rb	66.0538	236.742	0.27901	0.0001
P07147	5,6-dihydroxyindole-2-carboxylic acid oxidase	Tyrp1	5046.62	18320.4	0.27546	0.00016
Q7TPQ9	Arrestin domain-containing protein 3	Arrdc3	19.0149	69.8237	0.27233	0.00202
Q3UGA5	Tetraspanin 10	Tspan10	50.0502	183.874	0.2722	6.2E-05
Q8BTG6	SREBP regulating gene protein	Spring1	5.67738	21.5342	0.26364	0.00528
A0A2I3BRQ3	Inter-alpha trypsin inhibitor, heavy chain 3	Itih3	40.6496	155.799	0.26091	0.00147
A9CLV6	Axotomy induced glycoprotein 2	Serinc1	304.864	1177.56	0.25889	3.1E-05
Q99J93	Interferon-induced transmembrane protein 2	Ifitm2	64.8724	262.267	0.24735	0.00012
Q3UN36	Glycoprotein integral membrane 1	Ginm1	36.7537	152.623	0.24081	0.009
F6R6P2	Sarcoglycan, epsilon	Sgce	67.7932	288.951	0.23462	6E-06
A0A0N4SVU8	Transmembrane protein 176A (Fragment)	Tmem176a	56.3587	246.627	0.22852	0.00547
Q91VK4	Integral membrane protein 2C	Itm2c	109.886	494.545	0.2222	8.7E-07
A0A0R4J1C7	Microfibrillar-associated protein 3	Mfap3	33.3515	151.399	0.22029	0.00223
Q3UYL1	Ubiquitin-like protein	Ubl3	252.28	1233.98	0.20444	0.00214
Q8CFE6	Sodium-coupled neutral amino acid symporter 2	Slc38a2	235.425	1157.31	0.20342	4.7E-05
Q3UDL6	Amyloid-like protein 2	Aplp2	73.7625	383.288	0.19245	1E-05
Q2TA50	Melan-A	Mlana	341.368	1894.7	0.18017	2.9E-05
P40749	Synaptotagmin-4	Syt4	44.0191	265.614	0.16573	2E-05
A0A494BAA4	Nedd4 family interacting protein 1	Ndfip1	64.6429	436.11	0.14823	5.3E-05
O89051	Integral membrane protein 2B	Itm2b	57.5017	423.816	0.13568	8.4E-07
A0A2I3BQL3	Lysosomal-associated protein transmembrane 4B (Fragment)	Laptm4b	35.3112	321.192	0.10994	0.00188
D3YVM2	Transmembrane protein 59	Tmem59	153.122	1441.39	0.10623	0.00017

Q9R1Q6	Transmembrane protein 176B	Tmem176b	85.3376	886.929	0.09622	3.3E-06
Q60961	Lysosomal-associated transmembrane protein 4A	Laptm4a	16.0048	351.45	0.04554	0.00033

Table S5. Molecular docking analysis was performed using CB-Dock2 to evaluate the binding interactions between the tyrosinases and the following carboxylic acids: 3-phenyllactic acid, lactic acid, L-pyroglutamic acid, and malic acid. Tyrosinase (Mouse) and tyrosinase (*Agaricus*) were respectively sourced from the AlphaFold and PDB databases with accession number of P11344 and 2y9x.

Compounds	Enzymes	Binding energy	pi-pi	H-bond	Weak H-bond	Ionic interaction
3-Phenyllactic acid	Tyrosinase(Mouse)	-6.2	0	6	0	0
	Tyrosinase (*Agaricus*)	-6.6	0	5	1	0
Lactic acid	Tyrosinase(Mouse)	-4.4	0	2	1	0
	Tyrosinase (*Agaricus*)	-4.2	0	5	1	0
L-Pyroglutamic acid	Tyrosinase(Mouse)	-5.7	0	0	0	1
	Tyrosinase (*Agaricus*)	-5.4	1	2	1	0
Malic acid	Tyrosinase(Mouse)	-5.2	0	5	1	2
	Tyrosinase (*Agaricus*)	-5	0	4	0	2
